# Optimizing Graphene Oxide Interlayer Spacing for Zeolitic Imidazolate Framework‐8 Growth Toward Enhanced Shale Oil Separation

**DOI:** 10.1002/advs.202508544

**Published:** 2025-08-18

**Authors:** Hang Wang, Longyu Wang, Zhibang Liu, Yu Cheng, Wen Jiang, Xiaopeng Sun, Chuan‐De Wu

**Affiliations:** ^1^ Shandong Key Laboratory of Intelligent Energy Materials School of Materials Science and Engineering China University of Petroleum (East China) Qingdao 266580 P. R. China; ^2^ Henan Key Laboratory of Polyoxometalate Chemistry College of Chemistry and Molecular Sciences Henan University Kaifeng 475004 P. R. China

**Keywords:** membrane, optimizing interlayer spacing, petroleum fractionation, shale oil, ZIF‐8

## Abstract

Hydrocarbons derived from petroleum typically undergo energy‐intensive fractional distillations. Membrane separation technologies with high permeability and selectivity offer an energy‐efficient alternative. However, traditional membranes frequently encounter issues, such as swelling, deformation and diminished flux. Herein, quaternary ammonium salts are utilized to precisely control the interlayer spacing of graphene oxide (GO), facilitating the in situ growth of zeolitic imidazolate framework (ZIF‐8) to develop ZIF‐8‐GO membranes for shale oil separation. The ZIF‐8 nanoparticles serve to cross‐link the GO layers, effectively expanding the interlayer spacing and preventing swelling in organic solvents. The resultant ZIF‐8‐GO membrane achieves nearly complete rejection of n‐docosane in n‐hexane with a solvent permeance of 16.4 ± 0.8 L m^−2^ h^−1^ bar^−1^, which is 71 times higher than that of commercial membranes such as the PuraMem^®^ membrane (0.23 L m^−2^ h^−1^ bar^−1^), and surpasses previously reported membranes. The membrane is capable of concentrating hydrocarbons containing fewer than 20 carbon atoms and maintains its separation efficiency when transitioning from simple binary mixtures to more complex shale oil. This research opens a new avenue for the integration of MOF‐GO membranes into petroleum fractionation and provides a practical framework for the precise separation of shale oil.

## Graphical abstract

Quaternary ammonium salts are used to control the spacing between GO layers, enabling in situ growth of ZIF‐8. The resulting ZIF‐8‐GO membrane exhibits nearly complete rejection of n‐docosane in n‐hexane, solvent permeance of 16.4 ± 0.8 L m^−2^ h^−1^ bar^−1^, and enriches alkanes and aromatics with a molecular weight cut‐off of 310 g mol^−1^ in shale oil.

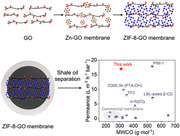

## 1 Introduction

Hydrocarbons are essential fuels and feedstocks for the production of chemicals and materials, playing a pivotal role in the chemical and petrochemical industries.^[^ ^1^ ^]^ However, hydrocarbons from petroleum are typically complex mixtures that rely on energy‐intensive distillation processes for separation and purification. These processes account for 10‐15% of global energy consumption, highlighting the urgent need for energy‐efficient alternatives.^[^ ^1^, ^2^ ^]^ Membrane‐based separation technologies have emerged as a promising solution, owing to their low energy requirements and operational simplicity.^[^ ^3^ ^]^ Despite their potential, polymeric membranes face heavy limitations, including swelling, irreversible aperture changes, and structural deformation under harsh conditions. These challenges undermine their permeability and selectivity, making it difficult to achieve high‐performance separation of hydrocarbons from petroleum.

Metal‐organic framework materials (MOFs), with their intrinsic porosity, high surface area, tunable pore sizes and versatile functionalities, have shown great promise in gas^[^ ^4^ ^]^ and liquid^[^ ^3^, ^5^ ^]^ separation applications. MOF membranes feature intrinsic cavities, well‐aligned structures and uniform pore size, providing additional transport pathways for small molecules and offering superior perm‐selective properties.^[^ ^6^ ^]^ Conventional fabrication methods often involve growing MOFs on porous substrates^[^ ^7^ ^]^ or incorporating MOF nanoparticles into polymer or graphene oxide (GO) matrix.^[^ ^3^, ^8^ ^]^ However, the aggregation of MOF nanoparticles in these systems frequently compromised membrane stability and reduced selectivity.^[^ ^9^ ^]^ Recent advances have shown that GO nanosheets with abundant functional groups can promote the in situ growth of nano‐sized MOFs, mitigating aggregation issues.^[^ ^3^, ^10^ ^]^ This approach not only enhances membrane stability but also compensates for defects, offering significant potential for challenging separations like petroleum fractionation.

**Figure 1:**
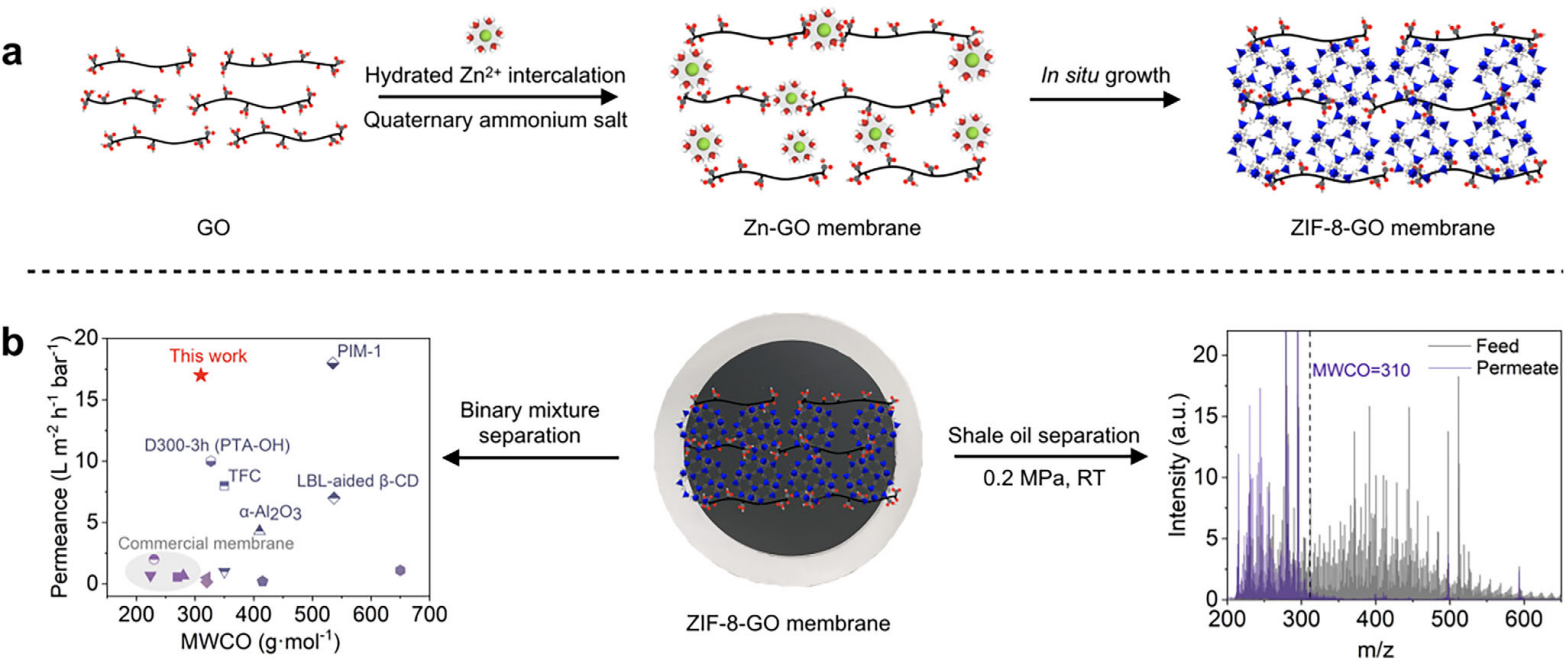
ZIF‐8‐GO membrane for shale oil separation. a) Schematic illustration of the synthesis process of ZIF‐8‐GO membranes. b) Performance comparison of the ZIF‐8‐GO membrane with reported membranes in binary system separation, along with FT‐ICR MS of ZIF‐8‐GO membrane of the feed and permeate of shale oil.

This work presents a novel ZIF‐8‐GO composite membrane, created using quaternary ammonium salts to regulate the interlayer spacing of GO membrane and promote the in situ growth of ZIF‐8 nanoparticles (**Figure** **1**a). This design creates rapid transport channels for organic solvents while maintaining high selectivity. The resulting membrane achieves nearly complete rejection of n‐docosane in n‐hexane solvent, with a solvent permeation rate of 16.4 ± 0.8 L m^−2^ h^−1^ bar^−1^ at room temperature, significantly surpassing the performance of reported membranes (Figure 1b). Furthermore, the ZIF‐8‐GO membrane demonstrates excellent stability and permeability during processing shale oil, with a flux of 17.4 ± 1.7 L m^−2^ h^−1^ bar^−1^ at 0.2 MPa. Advanced characterization using atmospheric pressure photoionization Fourier transform ion cyclotron resonance mass spectrometry (FT‐ICR MS) revealed that the permeate was enriched with alkanes and aromatics, with a molecular weight cut‐off (MWCO) of 310 g mol^−1^. This highlights the membrane's ability to selectively fractionate hydrocarbons in both simple binary systems and complex shale oil mixtures. These findings demonstrate the versatility and transformative potential of MOF membranes in modern hydrocarbon processing, offering a sustainable and scalable solution for energy‐efficient separations.

## 2 Results

### 2.1 Synthesis and Structural Characterization of ZIF‐8‐GO Membrane

**Figure 2:**
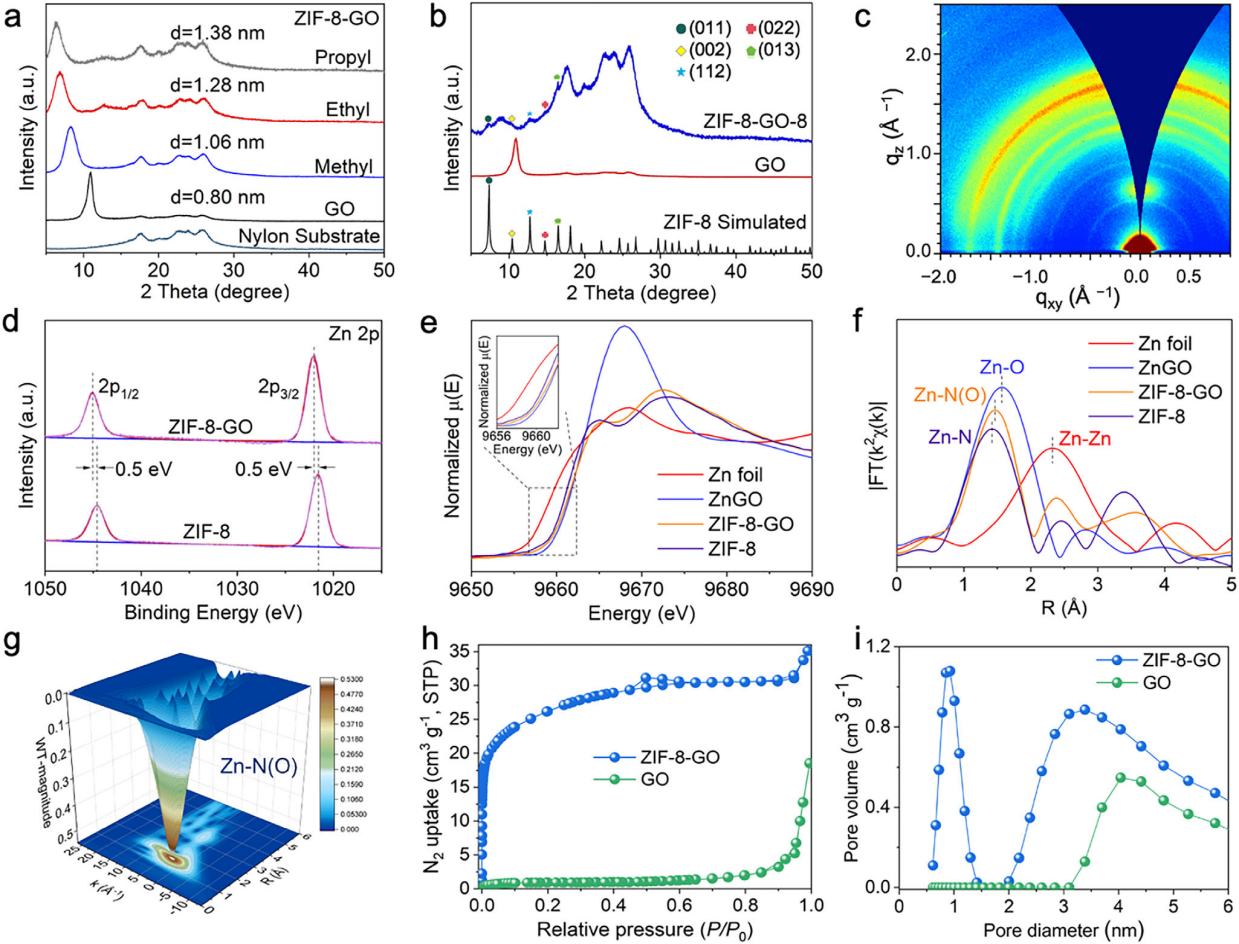
Characterizations of ZIF‐8 powder, GO and ZIF‐8‐GO membranes. a) PXRD patterns of GO, nylon substrate and ZIF‐8‐GO membranes with different inserted quaternary ammonium salt groups (methyl, ethyl and propyl). b) GIXRD patterns of GO and ZIF‐8‐GO membranes, and simulated PXRD pattern of ZIF‐8. c) GIWAXS pattern of ZIF‐8‐GO membrane. d) Zn 2p XPS spectra of ZIF‐8‐GO membrane and ZIF‐8. e) Zn K‐edge XANES spectra of Zn foil, ZnGO membrane, ZIF‐8‐GO membrane and ZIF‐8 powder. f) FT‐EXAFS of Zn K‐edge in Zn foil, ZnGO membrane, ZIF‐8‐GO membrane and ZIF‐8 powder. g) The 3D contour wavelet transform (WT) representation with 2D projection of EXAFS for ZIF‐8‐GO membrane. h) N_2_ sorption isotherms of GO and ZIF‐8‐GO membrane, and i) their corresponding pore size distributions.

In the present work, zinc ions (Zn^2+^) and quaternary ammonium salts were initially incorporated into the interlayers of GO to form a homogeneous ZnGO membrane. The membrane was subsequently subjected to vacuum‐assisted filtration using a dimethylimidazole aqueous solution to promote the in situ growth of ZIF‐8 nanocrystals within the GO interlayers.^[^ ^11^ ^]^ To demonstrate the role of quaternary ammonium salts in adjusting GO interlayer spacing, powder X‐ray diffraction (PXRD) analysis was performed. The results revealed a systematic increase in interlayer spacing, proportional to the molecular weight of the used quaternary ammonium salts (**Figure** **2**a). This expansion facilitated the growth of ZIF‐8 crystals within the confined GO channels, resulting in the formation of the ZIF‐8‐GO membrane.

Fourier‐transform infrared attenuated total reflection (FTIR‐ATR) spectroscopy was conducted to confirm the incorporation of GO and ZIF‐8 in the ZIF‐8‐GO membrane (Figure S1, Supporting Information). Characteristic peaks at 3135 and 421 cm^−1^ correspond to C–H stretching vibration in the imidazole ring and the Zn–N bond in ZIF‐8, respectively.^[^ ^12^ ^]^ The disappearance of the 1735 cm^−1^ peak indicated coordination between the carboxyl groups of GO and Zn^2+^ ions. Notably, the absence of peaks related to quaternary ammonium salts confirmed their removal during membrane preparation (Figure S2, Supporting Information). Raman spectra further implied that the defect‐to‐graphite band intensity ratio (I_D_/I_G_) increased with the molecular weight of the ammonium salts, indicating the formation of well‐structured channels beneficial for shale oil separation (Figure S3, Supporting Information).^[^ ^13^ ^]^ The interlayer adjustment could impact the membrane's separation performance, and the large interlayer structure could lead to an increase in flux and a decrease in retention rate. Therefore, the ZIF‐8‐GO membrane with tetramethylammonium chloride modified was first investigated. Grazing Incidence X‐ray diffraction (GIXRD) patterns were conducted to confirm the growth of ZIF‐8 in ZIF‐8‐GO membrane (Figure 2b). Meanwhile, the grazing‐incidence wide‐angle X‐ray scattering (GIWAXS) pattern of the ZIF‐8‐GO membrane became continuous diffraction rings with weakened intensity, indicating that ZIF‐8 in the ZIF‐8‐GO membrane was nonoriented and exhibited poor crystallinity (Figure 2c).^[^ ^14^ ^]^ UV–vis spectroscopy further validated the interaction between ZIF‐8 and GO (Figure S4, Supporting Information). A redshift in the characteristic peak of ZIF‐8 (202.8 nm) reflected strong interfacial interactions between the two components (Figure S4, Supporting Information),^[^ ^15^ ^]^ contributing to the membrane's structural integrity. Mechanical testing indicated that the ZIF‐8‐GO membrane possessed superior mechanical strength and flexibility to the GO membrane (Figure S5, Supporting Information).

X‐ray photoelectron spectroscopy (XPS) provided insights into the chemical states and bonding environment within the membranes. The presence of Zn and N elements in the ZIF‐8‐GO membrane from ZIF‐8, along with C and O from GO, was confirmed (Figure S6, Supporting Information). The XPS wide scan spectra indicated the absence of Cl^−^ associated with the quaternary ammonium salts, indicating quaternary ammonium salts were effectively removed (Figure S6, Supporting Information).^[^ ^16^ ^]^ Deconvolution C 1s spectra revealed peaks at 285.5 and 284.5 eV, corresponding to C–N and C–C bonds in 2‐methylimidazole (Figure S7, Supporting Information).^[^ ^17^ ^]^ In addition, the XPS spectra of ZIF‐8‐GO membrane showed additional components (C–O and O–C = O) associated with the carbonyl group of GO.^[^ ^18^ ^]^ The Zn 2p spectrum of ZIF‐8‐GO membrane exhibited a positive binding energy shift of 0.5 eV compared to that of ZIF‐8, indicating the electron cloud transfer from Zn atoms to O atoms in the ZIF‐8‐GO membrane (Figure 2d).^[^ ^18^, ^19^ ^]^ The N 1s XPS analysis of ZIF‐8‐GO membrane exhibited two deconvoluted peaks at 399.0 and 400.5 eV, attributed to Zn–N and N–H bonds, respectively. Importantly, the Zn–N bonds in ZIF‐8‐GO membrane demonstrated a positive shift of 0.4 eV compared to ZIF‐8 (Figure S8, Supporting Information), indicating a strong interaction between Zn^2+^ and the neighboring N atom.^[^ ^20^ ^]^

The coordination environment of Zn^2+^ in ZIF‐8‐GO membrane was further investigated by synchrotron radiation X‐ray absorption spectroscopy (XAS) and Fourier transform extended X‐ray absorption fine structure (FT‐EXAFS) (Figure 2e,f). The absorption energy near the K edge of Zn^2+^ in the ZIF‐8‐GO membrane was consistent with that of ZIF‐8, indicating that the ZIF‐8‐GO membrane primarily exhibited Zn‐N coordination (Figure 2e). The X‐ray absorption near edge structure (XANES) of Zn K edge in ZIF‐8‐GO membrane showed distinct shifts compared to ZIF‐8 and ZnGO, suggesting a stronger interaction between Zn and N in 2‐methylimidazole (Figure 2e). FT‐EXAFS provided short‐range local structure information around Zn (Figure 2f). A prominent peak at 1.47 Å, observed in ZIF‐8, ZnGO and ZIF‐8‐GO membranes, lied between the Zn–N (1.41 Å) and Zn–O (1.56 Å) first‐shell interaction distances, confirming the coexistence of Zn–N and Zn–O bonds. Wavelet transform (WT) analysis corroborated these observations, highlighting the role of Zn^2+^ as a structural bridge between GO and ZIF‐8 components (Figure 2g; Figure S9, Supporting Information).^[^ ^21^ ^]^ The Brunauer–Emmett–Teller (BET) specific surface area of ZIF‐8‐GO membrane (97 m^2^ g^−1^) was higher than that of GO (4 m^2^ g^−1^) due to the incorporation of ZIF‐8 (Figure 2h). Pore size distribution, calculated using nonlocal density flood theory (NLDFT), demonstrated the inheritance of ZIF‐8's microporous structure within the ZIF‐8‐GO membrane (Figure 2i; Figure S10 and Table S1, Supporting Information). These results demonstrated that Zn^2+^ successfully facilitated the integration of ZIF‐8 within GO layers, enabling confined crystal growth and the formation of a well‐structured ZIF‐8‐GO membrane.

**Figure 3:**
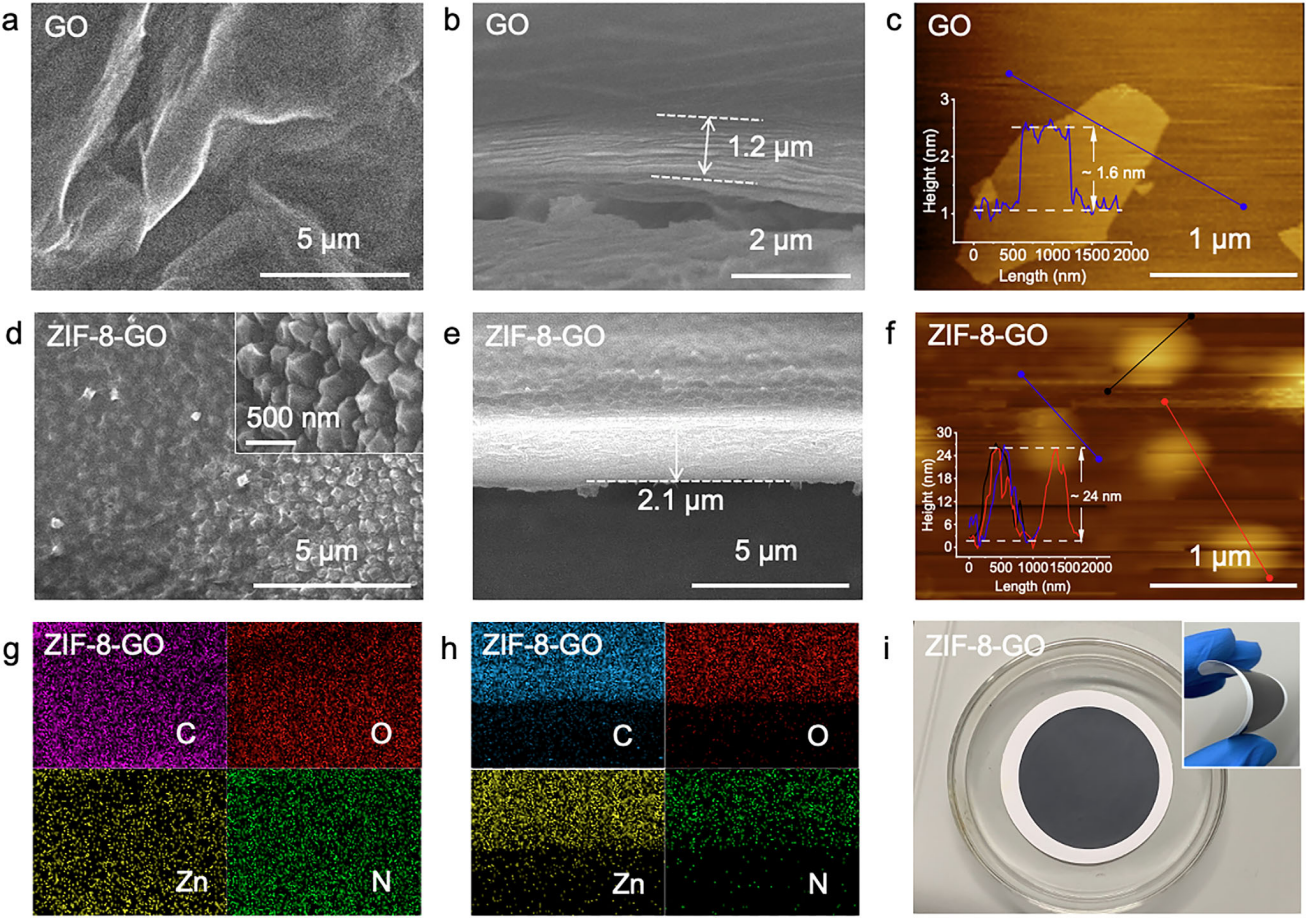
a) Top‐view SEM and b) cross‐section SEM images of GO membrane. c) AFM image of GO (inset corresponding height profile of GO). d) Top‐view SEM and e) cross‐section SEM images of ZIF‐8‐GO membrane. f) AFM image of ZIF‐8‐GO membrane (inset corresponding height profile of ZIF‐8‐GO). Energy dispersive X‐ray spectroscopy (EDS) elemental mapping images of g) C, O, Zn, and N in the top‐view and h) cross‐section of ZIF‐8‐GO membrane. i) Photograph of flat and curved (inset) ZIF‐8‐GO membranes.

The morphologies of GO and ZIF‐8‐GO membranes were investigated through field‐emission scanning electron microscopy (FE‐SEM) and atomic force microscopy (AFM). FE‐SEM images showed the wrinkled surface morphology and orderly layered structure of GO nanosheets, with a thickness of ≈ 1.6 nm (**Figure** **3**a–c). In contrast, ZIF‐8‐GO membrane exhibited a continuous ZIF‐8 layer on both the surface and inner layers of GO (Figure 3d), increasing the membrane thickness from 1.2 to 2.1 µm (Figure 3b,e). AFM images further confirmed the increased thickness of ZIF‐8‐GO membrane (≈24 nm), attributed to the incorporation of ZIF‐8 nanoparticles within the GO structure (Figure 3f). These findings highlight the successful integration of ZIF‐8 into GO, creating a robust and well‐defined composite structure. Energy dispersive X‐ray spectroscopy (EDS) mappings were used to characterize the elemental distributions (C, O, Zn, and N) within the ZIF‐8‐GO membrane. Uniform dispersion of these elements across the membrane surface and interlayers was observed (Figure 3g,h). The confined growth of ZIF‐8 within the GO interlayers limited excessive crystallization, which maintained the π‐π stacking effect between GO layers while expanding the membrane spacing and inheriting the crucial flexibility (Figure 3i). We further compared the in situ growth ZIF‐8 on GO under unconfined conditions, which led to a loss of membrane homogeneity (Figure S11, Supporting Information). This approach by using quaternary ammonium salts preserved the π‐π stacking interactions between GO layers while expanding the interlayer spacing, enhancing the membrane flexibility. Additionally, stability tests demonstrated no structural change in the ZIF‐8‐GO membrane after one month in polar (deionized water) and nonpolar (n‐hexane) solvents, as evidenced by retained PXRD patterns (Figures S12 and S13, Supporting Information). In comparison, GO membranes swelled and collapsed within two days in polar solvents due to drastic interlayer expansion (Figures S14 and S15, Supporting Information). Thermogravimetric analysis (TGA) showed that the decomposition temperature of the ZIF‐8‐GO membrane was mainly dominated by the GO component (Figure S16, Supporting Information). These results confirm that ZIF‐8‐GO membranes are robust and stable under various conditions, making them suitable for complex applications like oil separation.

### 2.2 Separation Performance and Mechanism of ZIF‐8‐GO Membrane

**Figure 4:**
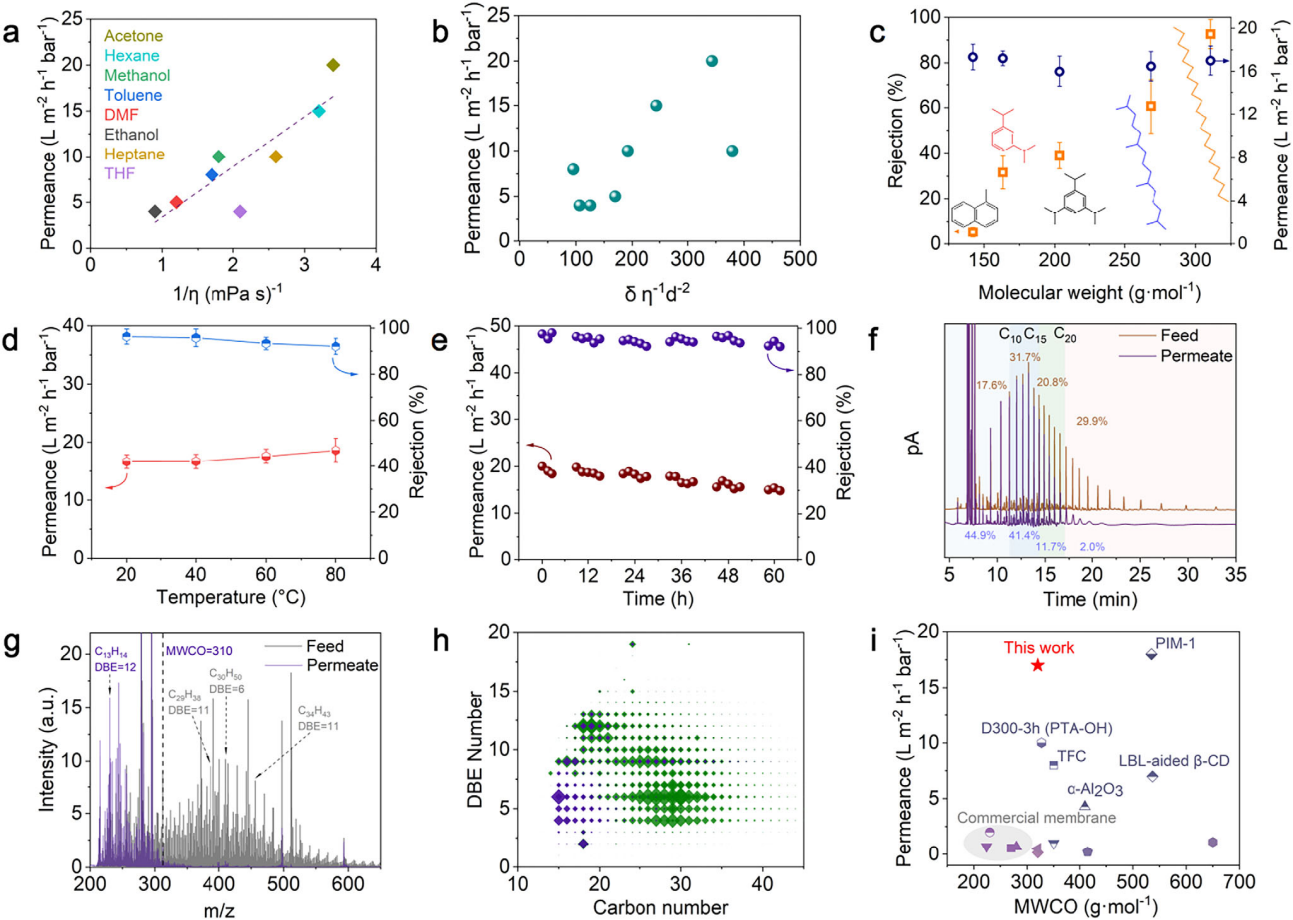
a) Polar and nonpolar solvent permeability as a function of the inverse viscosity. b) Inverse viscosity multiplied by molecular diameter and the inverse of Hansen's solubility parameter. c) Permeance and rejection of hydrocarbons from binary mixtures using ZIF‐8‐GO membranes. d) Permeance and rejection of n‐docosane solution at different temperatures by ZIF‐8‐GO membrane. e) Permeance and rejection of n‐docosane using ZIF‐8‐GO membrane within 60 h. f) Performance comparison of representative membranes and ZIF‐8‐GO membrane. g) Gas chromatography (GC) spectra of diluted shale oil feed and permeate solutions. h) FT‐ICR MS spectra of feed and permeate of diluted shale oil solutions. i) Double bond equivalent versus carbon number for the feed (green) and the permeate (purple) of diluted shale oil feed and permeate solutions.

The structural adaptability of ZIF‐8‐GO membranes enables precise fractionation of petroleum hydrocarbons. The ZIF‐8‐GO membrane, formed by ZIF‐8 crystals cross‐linking GO sheets, created a robust structure that facilitated the rapid transport of both polar and nonpolar solvents (**Figure** **4**a). Initial evaluations of GO membranes revealed no permeance to nonpolar solvents like n‐hexane. The ZIF‐8‐GO membrane demonstrated permeance inversely proportional to solvent viscosity, adhering to the Hagen‐Poiseuille law and indicating size‐selective separation (Figure 4a).^[^ ^22^ ^]^ Correlations involving the inverse of viscosity, Hansen solubility parameters and molecular diameters further highlighted a stronger solution‐diffusion transport mechanism (Figure 4b; Figure S17 and Table S2, Supporting Information).^[^ ^23^ ^]^ Although there is no designated solvent or solute in complex petroleum mixture fractionation, the chemical composition of petroleum contains a variety of hydrocarbon and non‐hydrocarbon compounds.^[^ ^24^ ^]^ The ZIF‐8‐GO membranes were evaluated for filtration performance with highly nonpolar systems, specifically hydrocarbon solutions in n‐hexane at 500 ppm (Figure 4c). Unlike GO membranes, ZIF‐8‐GO membranes did not compress under filtration due to the reduced number of carboxyl groups replaced by metal ions.^[^ ^25^ ^]^ The flux of the ZIF‐8‐GO membrane increased with filtration pressure in a dead‐end filtration setup (Figure S18, Supporting Information), demonstrating excellent permeability and structural integrity under pressures up to 0.25 MPa (Figure S19, Supporting Information). After 3 h of continuous operation, the ZIF‐8‐GO membrane achieved stable permeability of 17.5 ± 1.2, 17.2 ± 0.7, 16.4 ± 1.3, 16.7 ± 1.3 and 16.4 ± 0.8 L m^−2^ h^−1^ bar^−1^ at 0.2 MPa for methylnaphthalene, 1,3‐diisopropylbenzene, 1,3,5‐triisopropylbenzene, pristane and n‐docosane (Figure 4c), respectively. The ZIF‐8‐GO membrane achieved nearly complete rejection of n‐docosane in n‐hexane with a solvent permeance of 16.4 ± 0.8 L m^−2^ h^−1^ bar^−1^, which is 71 times higher than that of commercial membranes such as the PuraMem^®^ membrane (0.23 L m^−2^ h^−1^ bar^−1^). Molecular dynamics (MD) simulations displayed the attractive interaction between alkane molecules and the ZIF‐8‐GO membrane arose from van der Waals and electrostatic interactions. The n‐docosane could not permeate through the nanoconfined channels of the ZIF‐8‐GO membrane, instead adsorbing onto the ZIF‐8‐GO surface (Figure S20, Supporting Information). The adsorption of n‐docosane on the ZIF‐8‐GO surface was evidenced by the initial slope and subsequent plateau (Figure S20, Supporting Information), indicating surface adsorption without diffusion. In contrast, the linear permeation profile of hexane demonstrated its effective diffusion through the ZIF‐8‐GO nanochannels due to its smaller kinetic diameter (Figure S21, Supporting Information). The impact of quaternary ammonium salts and GO content on membrane performance was also investigated (Figures S22 and S23, Supporting Information). Larger ammonium salts expanded the GO interlayer spacing, significantly increasing n‐hexane flux from 16.4 to 37.0 L m^−2^ h^−1^ bar^−1^ (Figure 2a; Figure S24, Supporting Information). However, this expansion reduced the retention of n‐docosane to 9.8% due to defects caused by larger ZIF‐8 particles and excessively wide interlayer spacing (Figures S3 and S24, Supporting Information). Increasing GO content improved retention but reduced solvent permeability, as greater channel curvature hindered transport (Figure S23, Supporting Information).^[^ ^26^ ^]^ At elevated temperatures, the ZIF‐8‐GO membrane demonstrated enhanced n‐hexane permeance, reaching 19.5 ± 1.4 L m^−2^ h^−1^ bar^−1^ at 80 °C due to the reduced solvent viscosity,^[^ ^27^ ^]^ and the rejection of n‐docosane remained above 90% (Figure 4d). Long‐term tests confirmed the membrane's stability, maintaining constant permeance under 0.2 MPa for 60 h without structural collapse (Figure 4e). The XRD patterns of ZIF‐8‐GO membrane remained unchanged before and after the 60 h stability test (Figure S25, Supporting Information), confirming structural integrity. These findings demonstrate the exceptional efficiency, structural robustness and scalability of ZIF‐8‐GO membranes, highlighting their potential as advanced materials for hydrocarbon processing.

### 2.3 Shale Oil Separation Performance Evaluation

To the best of our knowledge, MOF membranes haven't been widely reported for efficient selective separation of shale oil specifically. The ZIF‐8‐GO membranes were further tested with a real shale oil sample from the Gulong shale oil reservoir in the northern Songliao Basin (Daqing Oilfield) to evaluate their separation performance. The shale oil was diluted with n‐hexane to a concentration of 2000 ppm, and processed using a homemade dead‐end filtration device. The ZIF‐8‐GO membrane demonstrated stable permeability, achieving 17.4 ± 1.7 L m^−2^ h^−1^ bar^−1^ at 0.2 MPa. Gas chromatography (GC) showed extremely high enrichment in the permeate, with up to 98% of hydrocarbons below C20 (molecular weight ≈ 310 g mol^−1^) being retained (Figure 4f). Retention times for various carbon numbers in the shale oil gas chromatogram were calibrated using a standardized normal saturated alkane solution (Figure S26, Supporting Information). The feed and permeate were further analyzed using a UPC_SIMDIS full range simulated distillation system. Hydrocarbons within the C10‐C15 range accounted for 41.4%, those in the C15 to C20 range contributed 11.7%, and those above C20 were only 2% of the permeate (Table S3, Supporting Information). FT‐ICR MS of the diluted shale oil feed and the corresponding permeate revealed a shift in the maxima of the most abundant fraction from 250–600 g mol^−1^ to below 310 g mol^−1^, confirming the enrichment of lower molecular weight hydrocarbons (Figure 4g). Additionally, components with carbon numbers below C20 accounted for a higher proportion, which is associated with lubricating oil and kerosene fuel (Figure 4g).^[^ ^22^ ^]^ The critical molecular weight cut‐off for the membrane, determined from mixed component retention, ranged from 280 to 310 g mol^−1^. The permeate contained not only straight‐chain alkanes but also aromatic hydrocarbons with higher double bond equivalent (DBE) values (Figure 4h). Compared to the commercial membranes and other systems reported in the literature, the ZIF‐8‐GO membrane can sufficiently reject (MWCO > 310 g mol^−1^) hydrocarbons in shale oil while keeping the flux higher than 16 L m^−2^ h^−1^ bar^−1^. (Figure 4i; Table S4, Supporting Information).^[^ ^22^, ^23^, ^28^ ^]^ These results highlight the ZIF‐8‐GO membrane's ability to effectively separate complex mixtures, transitioning seamlessly from binary solutions to dilute multicomponent systems, reinforcing its versatility and efficiency for advanced hydrocarbon processing.

## 3 Conclusion

In summary, this study introduces a novel approach to fabricate ZIF‐8‐GO membranes through vacuum‐assisted filtration and in situ confined growth. This membrane enables efficient separation of specific carbon fractions from shale oil. The interlayer channels of the GO framework, combined with the precise micropores of ZIF‐8, enable selective separation based on diffusion and adsorption mechanisms, distinguishing between paraffin and aromatics. Hydrocarbon separation results demonstrated that the ZIF‐8‐GO membrane maintains its advantages even when processing diluted mixtures containing thousands of components. This work offers a promising pathway for the construction of MOF‐hybridized GO membranes, delivering high solvent permeability and exceptional selectivity for shale oil separation. It offers a potential solution to critical challenges in modern hydrocarbon processing.

## Conflict of Interest

The authors declare no conflict of interest.

## Supporting information



Supporting Information

## Data Availability

The data that support the findings of this study are available in the supplementary material of this article.
