# Supplementary material for: Optimizing Graphene Oxide Interlayer Spacing for Zeolitic Imidazolate Framework‐8 Growth Toward Enhanced Shale Oil Separation
Source: Adv Sci (Weinh). 2025 Aug 18;12(43):e08544. doi: 10.1002/advs.202508544 (PMC12631834; doi:10.1002/advs.202508544)
Supplement: Supplementary file 1 — Supporting Information [file ADVS-12-e08544-s001.docx]

Supporting Information

Optimizing Graphene Oxide Interlayer Spacing for Zeolitic Imidazolate Framework-8 Growth toward Enhanced Shale Oil Separation

*Hang Wang, Longyu Wang, Zhibang Liu, Yu Cheng, Wen Jiang, Xiaopeng Sun, Chuan-De Wu**

H. Wang, L. Wang, Z. Liu, Y. Cheng, W. Jiang, C. Wu

Shandong Key Laboratory of Intelligent Energy Materials, School of Materials Science and Engineering, China University of Petroleum (East China), Qingdao 266580, P. R. China

E-mail: [cdwu@upc.edu.cn](mailto:cdwu@upc.edu.cn)

X. Sun

Henan Key Laboratory of Polyoxometalate Chemistry, College of Chemistry and Molecular Sciences, Henan University, Kaifeng 475004, P. R. China

**Materials**

Zn(NO_3_)_2_·6H_2_O (ALADDIN Reagent Co., Ltd.), ethanol (Beijing Chemical Reagent Company), 2-methylimidazole (Hmim, Energy Chemical Co., Ltd.), Graphite (Qingdao Everlasting Benefit Graphite Co,. Ltd.), H_2_SO_4_ (95%–98%, Sinopharm Chemical Reagent Co., Ltd.), KMnO_4_ (Sinopharm Chemical Reagent Co., Ltd.), H_2_O_2_ (30%, Sinopharm Chemical Reagent Co., Ltd.), HCl (36%–38%, Sinopharm Chemical Reagent Co., Ltd.), liquid nitrogen (Qingdao Xinke Chemical Technology Co., Ltd.), ylon 66 (Delvstlab), tetramethylammonium chloride (methyl can be changed by ethyl and propyl) (Energy Chemical Co., Ltd.) deionized water was produced by using the Ulupure purification system. All chemicals and solvents were used as received without further purification.

Instrumentation

The Powder X-ray diffraction patterns (PXRD) were recorded on a TD-3500 X-ray diffractometer (Dandong Tongda Science & Technology Co., Ltd.) with Cu-Kα radiation (λ = 1.54056 Å). The PXRD patterns were recorded in the range of 3º to 50º (2θ)。The sampling time was 0.2 s, and the spectrum scanning was completed under continuous scanning, biaxial linkage, operating voltage of 40 kV and a current of 30 mA.

Grazing incidence X-ray diffraction (GIXRD) patterns were also performed on TD-3500 X-ray diffractometer, only utilizing high-resolution parallel beams and different parameters. With other conditions unchanged, the sampling time was adjusted to 1 s and the scanning mode was changed to step scanning.

Fourier transform infrared attenuated total reflection (FTIR-ATR) spectra were measured on a Bruker ALPHA spectrometer at the range of 400-4000 cm^−1^.

Raman spectroscopy was performed using a HORIBA Scientific LabRAM HR Evolution spectrometer, which was outfitted with a 532 nm diode laser and a 50X objective lens (8 mm focal length). The spectrometer has a focal length of 800 mm and achieves a spectral resolution of approximately ~1 cm^−1^. Prior to analysis, the system underwent calibration using an internal silicon sample featuring a distinctive band at 520 cm^−1^.

Shimadzu UV-2700i UV-Vis Spectrophotometer recorded the UV absorption of materials in the 190-350 nm range.

N_2_ sorption isotherms were acquired at 77 K nitrogen atmosphere (99.999%) on BUILDER Kubo-X1000 after pretreating samples by heating them in vacuum to 120 °C and holding for 12 h. The pore size distributions were calculated by non-local density functional theory (NLDFT).

The surface chemical composition and state of the samples were analyzed by X-ray photoelectron spectroscopy (XPS) tests using a Thermo Fischer ESCALAB 250 Xi^+^ XPS system. The vacuum of the analysis chamber was 3×10^-9^ mbar, the excitation source was Al ka-rays (1486.68 eV), the operating voltage was 15 kV, the filament current was 10 mA, and the signals were accumulated for six cycles. Passing-Energy was tested at 50ev with a step size of 0.05 eV, and the energy standard was C1s=284.80 eV binding energy for charge correction.

X-ray absorption fine structure (XAFS) spectroscopy was carried out using the *Rapid XAFS* 2M (Anhui Absorption Spectroscopy Analysis Instrument Co., Ltd.) by transmission mode at 20 kV and 30 mA, and the Si (733) spherically bent crystal analyzer with a radius of curvature of 500 mm was used for Zn. The acquired XAS data were processed according to the standard procedures using the ATHENA module of Demeter software packages. The EXAFS spectra were obtained by subtracting the post-edge background from the overall absorption and then normalizing with respect to the edge-jump step. Subsequently, the χ(k) data were Fourier transformed to real (R) space using a hanning window (dk = 1.0 Å^−1^) to separate the EXAFS contributions from different coordination shells.

Field-emission scanning electron microscope (FE-SEM) images were recorded on a Hitachi Regulus 8100 scanning electron microscope with a voltage of 15 kV and a current of 10 μA. To enhance image quality, samples were sputter-coated with a gold layer to improve electrical conductivity prior to imaging. Elemental mappings were obtained using energy-dispersive X-ray spectroscopy (EDX) on the same Hitachi Regulus 8100 SEM, operating at 200 kV.

Thermogravimetric analysis (TGA) of activated samples (~5 mg) was performed using a Netzsch STA449F5 instrument with a temperature range of 40 °C-1000 °C, N_2_ atmosphere, and a heating rate of 10 °C min^−1^.

Atomic force microscopy (AFM) measurements were taken by Dimension ICON (Bruker, USA). An ethanol solution comprising the samples was dropped on a silicon substrate for AFM measurements. All images were captured by tapping mode with a scan rate at 1-2 Hz and 256 × 256-pixel resolution.

Gas chromatography (GC) was performed on a FULI Instrument GC9790 Plus gas chromatograph with a flame ionization detector (FID). At 320 ℃, 1 μL of permeate oil sample was injected into the split port at a split ratio of 20: 1. PONA chromatographic column (50m × 250μm × 0.5μm) was used for separation. The feed and permeate were further analyzed using a UPC_SIMDIS full range simulated distillation systems and fourier transform ion cyclotron resonance mass spectrometry (FT ICR MS, Thermofisher orbitrap fusion MS).

The mechanical properties of membranes were investigated using an Instron 5843 system (England). The membranes were cut into 2 cm × 0.5 cm and released along their length at a constant speed of 2 mm min^−1^ by using a 10 N sensor.

The crystallinity of ZIF-8-GO membrane was determined by grazing-incidence wide-angle X-ray scattering (GIWAXS, Xenocs XEUSS).

Experimental methods

*Synthesis of GO*

GO was prepared by oxidation of natural graphite powder according to the modified Hummers’ method.^[1]^

*Synthesis of GO membrane*

GO membrane was prepared by vacuum assisted filtration device. Initially, 4 mg of GO was dispersed in 10 mL of deionized water, followed by sonication for 60 minutes to achieve a uniform GO suspension. The suspension was then evenly coated onto a Nylon 66 substrate with a pore size of 0.45 µm under a vacuum pressure of 0.1 MPa. After drying at room temperature, the GO membrane was further treated in an oven at 60 degrees Celsius for 12 hours to complete its formation.

*Synthesis of ZIF-8*

ZIF-8 nanoparticles were prepared according to the previous literatures.^[2]^ Typically, a solution of Zn(NO_3_)_2_·6H_2_O (0.75 g, 2.5 mmol) and Hmim (1.68 g, 20 mmol) were dissolved in 50 mL of methanol and sonicated well. Then, the above two solutions were mixed and stirred at room temperature for 3 h. Subsequently, the mixed solution was centrifuged to obtain a white powder, which was washed repeatedly with methanol and dried in a vacuum oven at 60 ℃. Finally, the dried ZIF-8 was activated for 12 h at 120 ℃ under vacuum.

*Synthesis of ZnGO membranes*

10 mL of 0.4 mg mL^−1^ GO suspension was ultrasonicated for 30 min. Then, 0.05 mmol of Zn(NO_3_)_2_⋅6H_2_O were added to 10 mL of deionized water and then added to the GO suspension, and the mixture was sonicated for 30 min to obtain a colloidal suspension of ZnGO. After that, the obtained ZnGO colloidal suspension was filtrated on a Nylon 66 substrate under a vacuum to form a ZnGO membrane, and dried at 60°C.

*ZIF-8 directly in situ grown in GO membrane*

5 mL 0.4 mmol 2-methylimidazole was added to the ZnGO colloidal suspension and then ultrasonicated for 15 min to form the ZIF-8/GO suspension. The ZIF-8/GO membrane was finally obtained through vacuum-assisted filtration and dried under vacuum at room temperature.

*Synthesis of ZIF-8-GO membranes*

First, a 10 mL suspension of GO with a concentration of 0.4 mg mL^−1^ was prepared and sonicated for 1 hour to ensure proper dispersion. Following this, 0.05 mmol of Zn(NO_3_)_2_·6H_2_O was added and sonicated for 30 minutes to disperse it evenly. Subsequently, 0.4 mg quaternary ammonium salts with different groups (methyl, ethyl, and propyl) were introduced into the solution with vigorous stirring, after which 600 μL of ammonium hydroxide (NH_3_·H_2_O) was added and sonicated for 1 hour to achieve homogeneity. The resulting solution was then passed through a Nylon 66 substrate membrane using a vacuum-assisted filtration device. After the solution had traversed the substrate, the vacuum was maintained at room temperature for 60 minutes. Following this, 20ml deionized water and 50 mL of an aqueous solution containing 1.83 mmol of Hmim solution was added to the filtration device. The membrane was obtained once the solution completely passed through the substrate.

The membrane was then removed and air-dried overnight at room temperature, followed by placement in a 70°C oven for 10 hours to yield the ZIF-8-GO membrane.

*Membrane performance*

Membrane filtration performance was evaluated using a dead-end filtration device pressurized with nitrogen, with an effective membrane area of 3.14 cm^2^ and a pressure range of 0.5-2.5 MPa during the experiments. The permeability of different solvents through a membrane at a fixed pressure was determined by the time and volume of passage through a unit area of membrane, which was evaluated using Equation (1):

$F=\frac{V}{A\cdot\Delta t\cdot\Delta P}$ (1)

where *V* (L) is the penetration volume of the solvent, *A* (m^2^) is the effective filtration area, *Δt* (h) is the filtration time, and *ΔP* is the applied pressure.

The membrane retention of different organic molecules was evaluated by injecting 15 ml of feed solution into a dead-end filtration device. The rejection (*R*) was evaluated using equation (2):

$R=\left( 1-\frac{C_{p}}{C_{f}} \right)\times100\%$ (2)

where *C_p_* is the solute concentration in the permeate, and *C_f_* is the solute concentrations in the feed solution, which can be measured by ultraviolet spectrophotometer and gas chromatography.

*Hydrocarbon mixture separation*

The simulated oil feedstock with 5 solutes (500 ppm) dissolved in n-hexane mixture was filtered by a dead-end filter at 0.2 MPa. The rejection of solute was determined by gas chromatography. Discard the first 5 minutes of filtrate, and then collect the filtrate every 15 minutes thereafter to calculate the retention rate. The stabilized value will be the final result.

*Shale oil fractionation*

The feed and permeate were analyzed using GC with a UPC_SIMDIS full range simulated distillation systems, Fourier transform ion cyclotron resonance mass spectrometry (FT ICR MS, Thermofisher orbitrap fusion MS). was used to analyze the feed and permeate fluids of shale oil diluted with n-hexane. The ionization source was set as H-ESI electrospray ionization source, the scanning type was MS scanning, and the detector was Orbitrap, with an orbitrap resolution up to 5000k and a scanning range of 100 – 1000 Da; the radiofrequency (RF) lens was adjusted to 60%, and the AGC target was 1e6, with a maximum injection time of 100 ms, and the negative ion spray voltage was 2600 V. The sheath gas flow rate 5 arb, auxiliary gas flow rate 2 arb, sweeping gas flow rate 0.1 Tests were carried out at an ion transfer tube temperature of 300°C and an evaporator temperature of 20 °C at sheath gas, aux gas, and sweep gas flow rates of 5, 2, and 0.1 arb, respectively. Relationship between the number of double bond equivalents and carbon number in hydrocarbon compounds in feed and permeate fluids analyzed using Xcalibur 2.0.

*Calculation process*

All the molecular dynamics were performed using the Large-scale Atomic/Molecular Massively Parallel Simulator (LAMMPS)^[3]^ package. Periodic boundary conditions were applied in all three directions. The ZIF-FF force field^[4]^ and the Dreiding force field were adopted to model the interatomic interactions within the ZIF-8 framework and graphene oxide, respectively. The non-bonded interactions were described by the Lennard-Jones (LJ) potential and electrostatic interactions. The non-bonded potential parameters were obtained from the Direiding force field. For the electrostatic, atomic charges were assigned by the Qeq method^[5]^, and the coulombic terms were calculated using the particle-particle-particle-mesh (PPPM) algorithm. In the simulations, the ZIF-8-GO membrane consisting of ZIF-8 and graphene oxide was first relaxed under the NPT ensemble at 300K. After that, n-hexane and alkane were introduced, and the separation simulations were performed within the NVT ensemble with a timestep of 1fs.

**Supplementary figures**

**
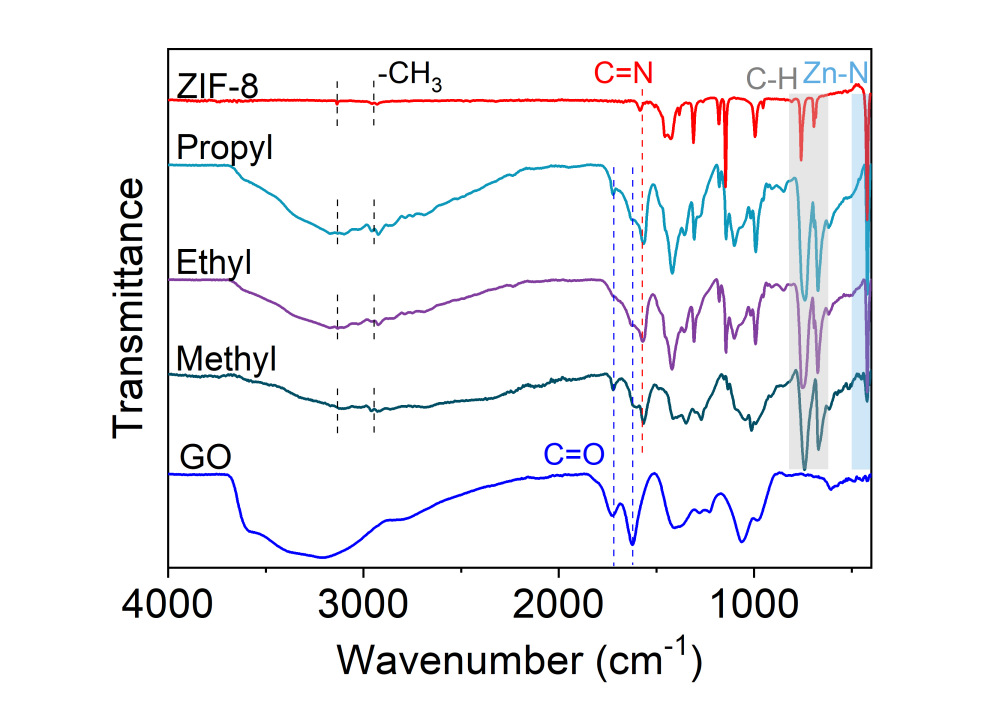
**

**Figure S1.** FT-IR spectra of GO, ZIF-8 and quaternary ammonium salts with different groups (methyl, ethyl and propyl) inserted ZIF-8-GO membranes.


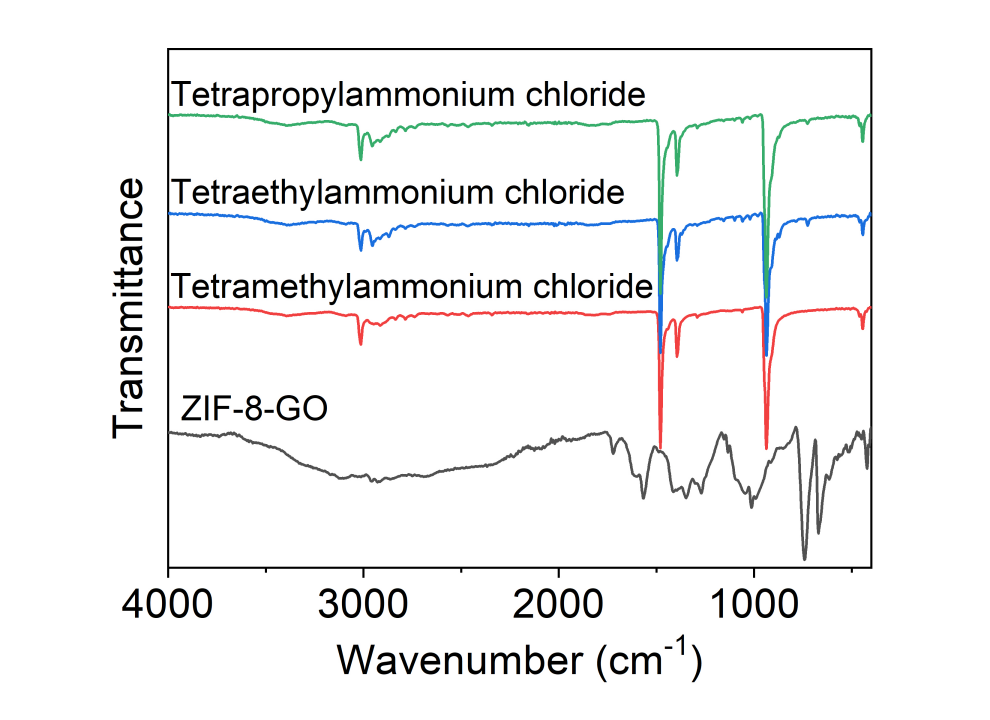


**Figure S2.** FT-IR spectra of ZIF-8-GO modified with tetramethylammonium chloride and FT-IR spectra of quaternary ammonium salts with different groups (methyl, ethyl and propyl).

**
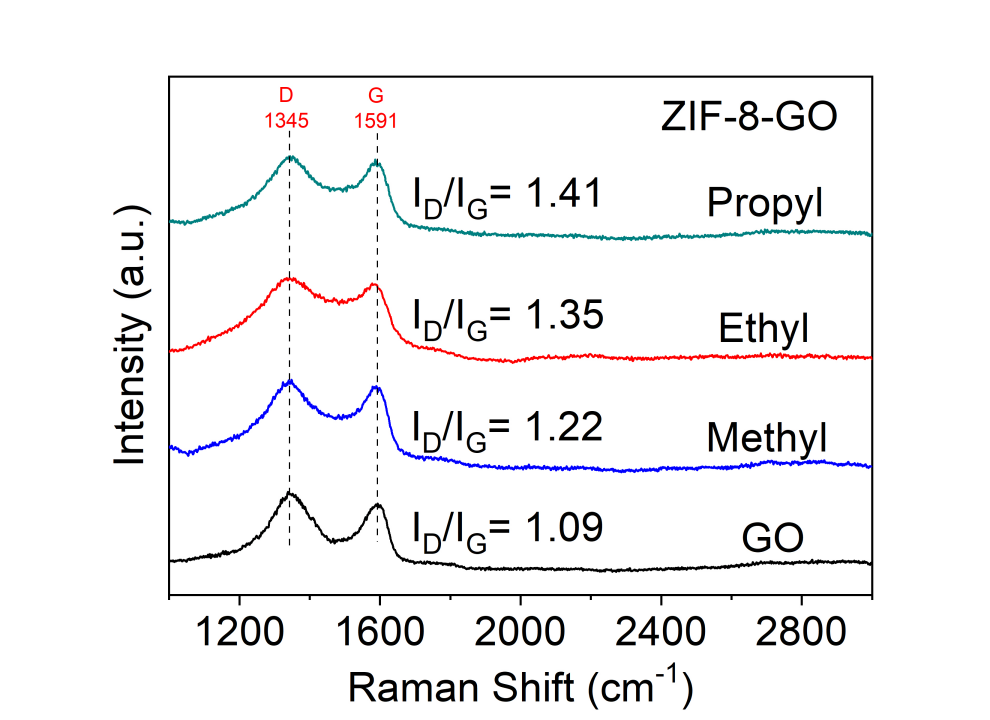
**

**Figure S3.** Raman spectra of GO and quaternary ammonium salts with different groups (methyl, ethyl and propyl) inserted ZIF-8-GO membranes.


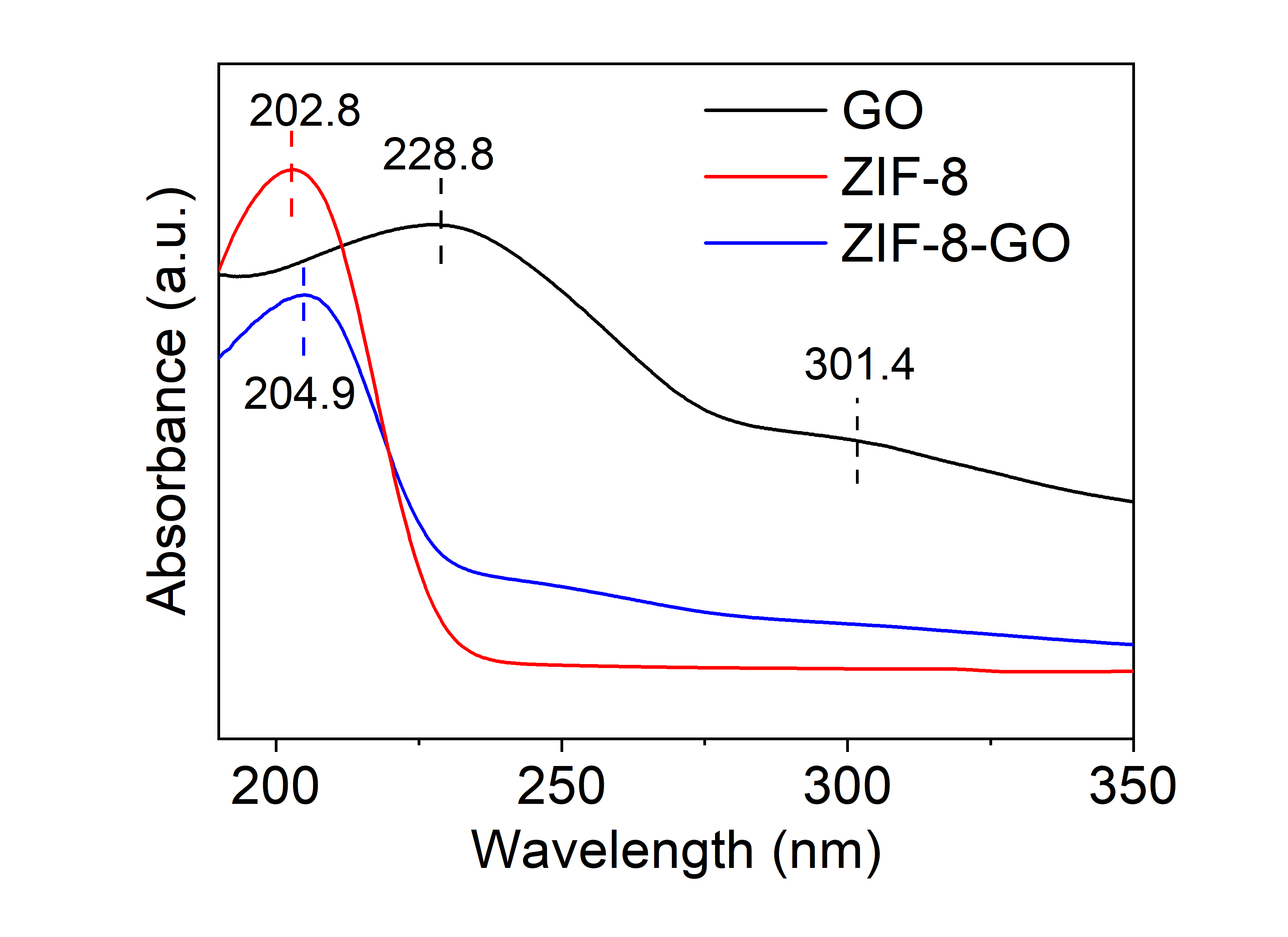


**Figure S4.** UV–vis spectra of ZIF-8 powder, GO and ZIF-8-GO membranes.


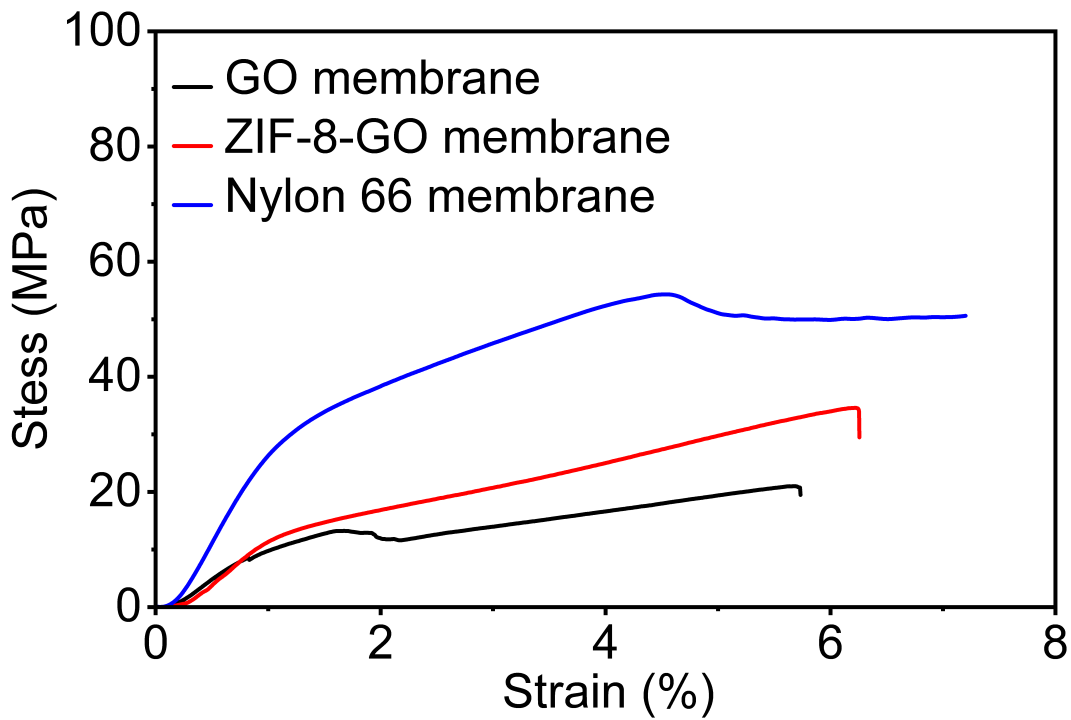


**Figure S5.** The stress-strain curves of GO, ZIF-8-GO and nylon 66 membrane.

**
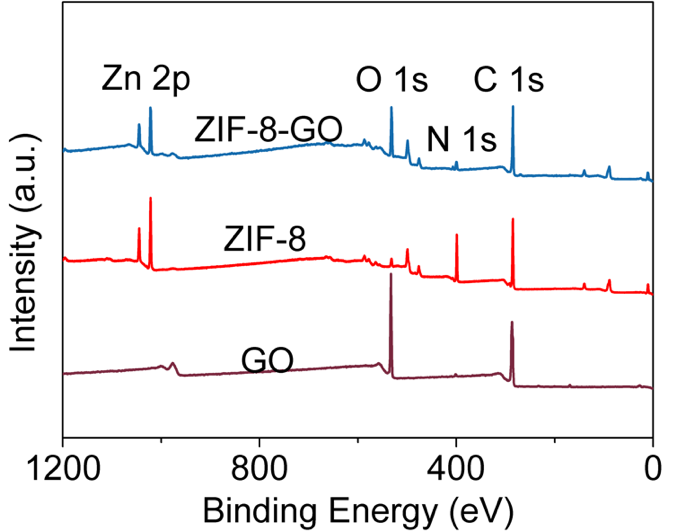
**

**Figure S6.** The full range XPS spectra of GO, ZIF-8 and ZIF-8-GO membrane.


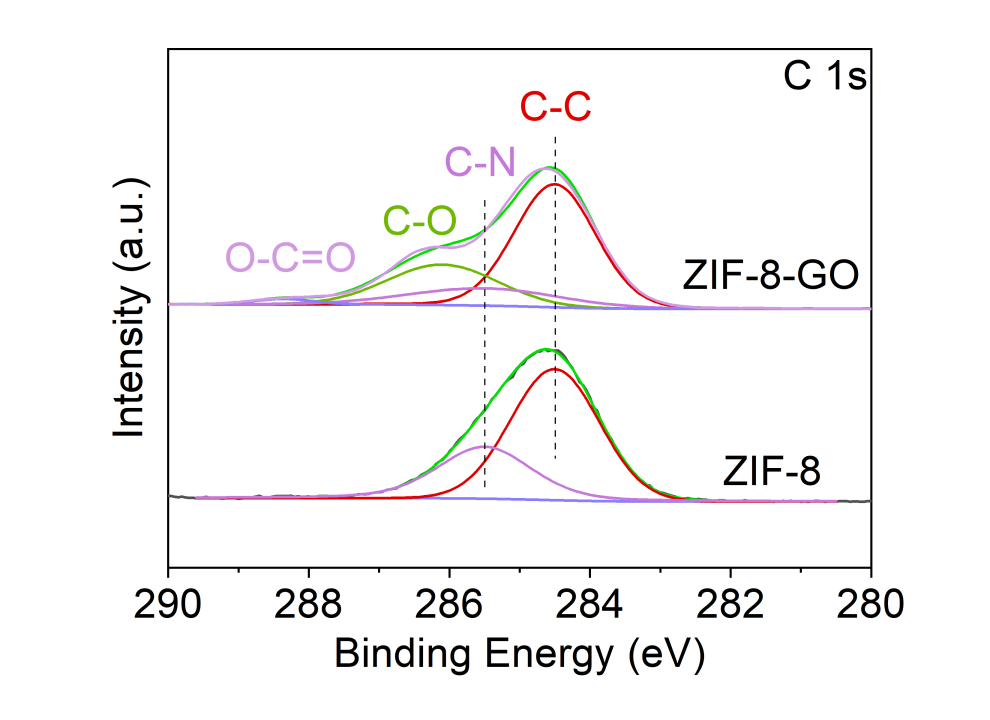


**Figure S7.** C 1s XPS spectra of ZIF-8 and ZIF-8-GO membrane.


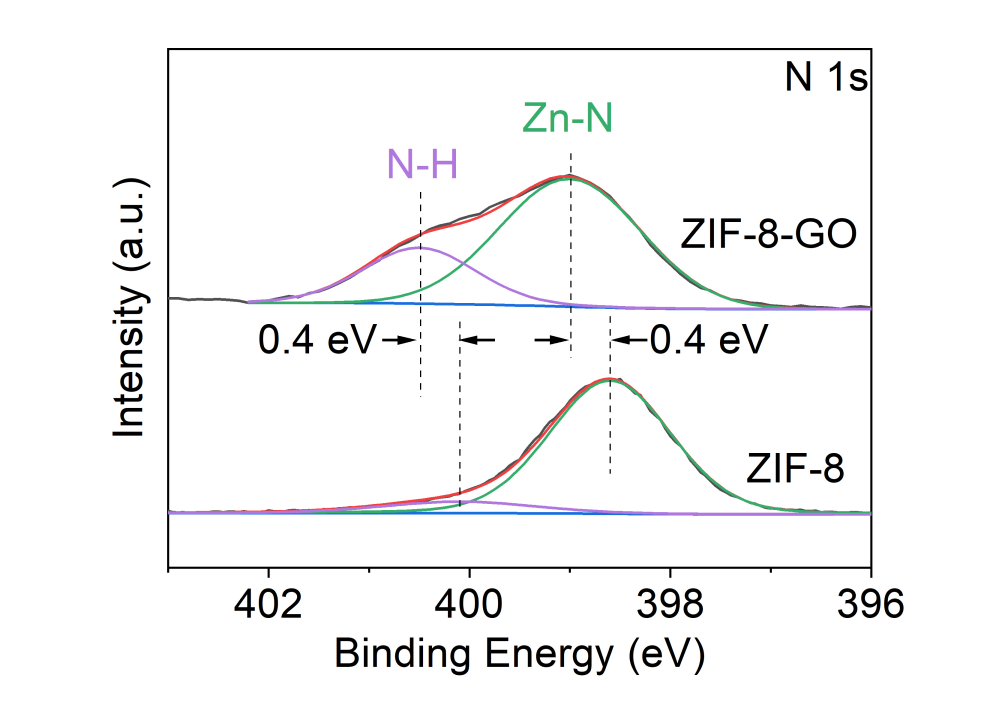


**Figure S8.** N 1s XPS spectra of ZIF-8 and ZIF-8-GO membrane.


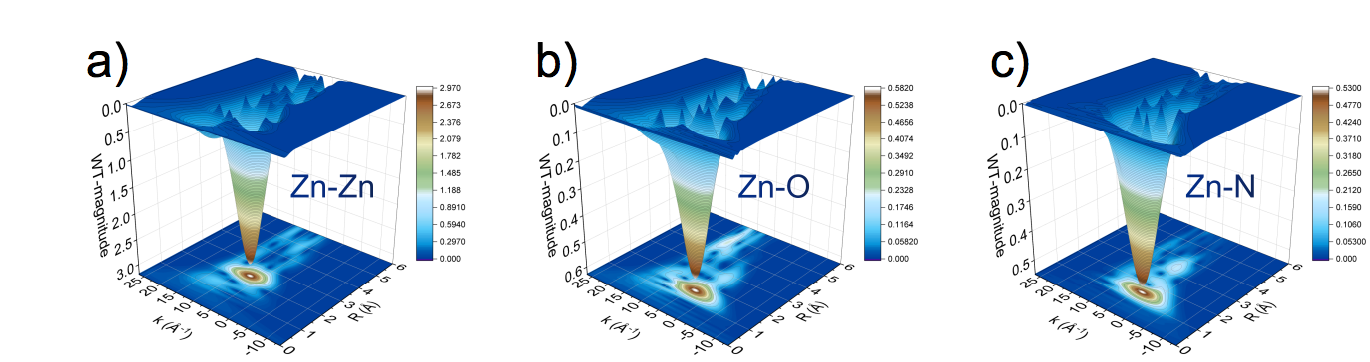


**Figure S9.** The 3D contour wavelet transform (WT) representation with 2D projection of EXAFS for a) Zn foil, b) ZnGO membrane and c) ZIF-8.

**
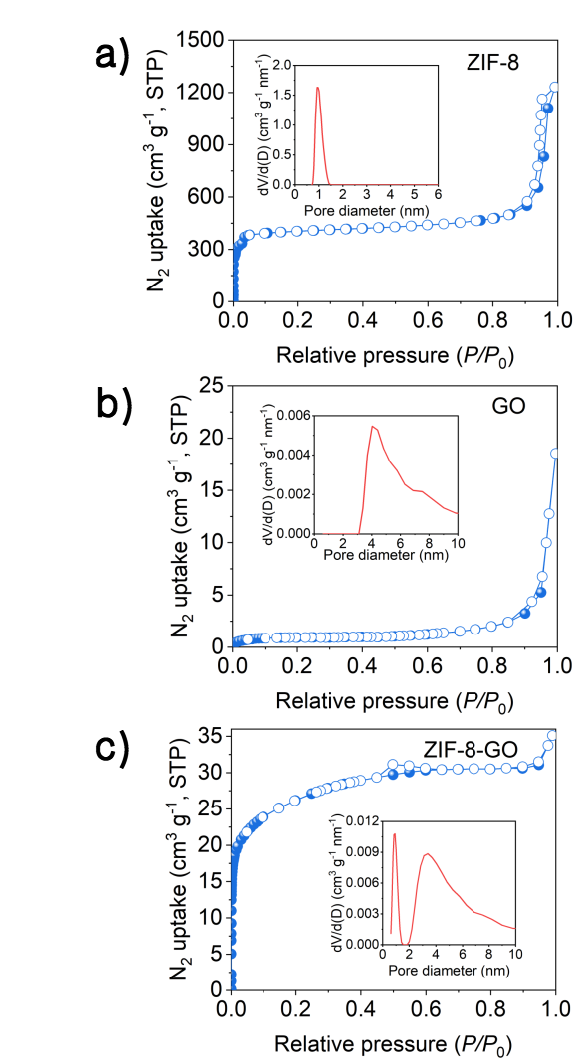
**

**Figure S10.** a) N_2_ sorption isotherms and pore size distributions (inset) of ZIF-8, b) GO and c) ZIF-8-GO membrane.


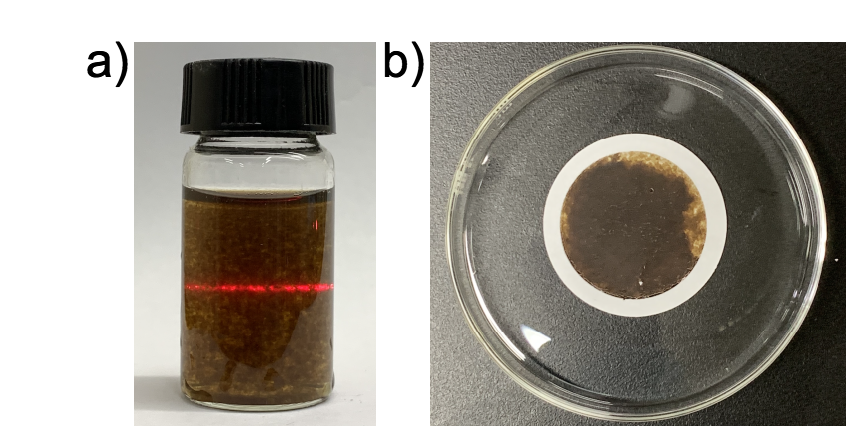


**Figure S11.** Photographs of a) ZIF-8 mixed GO colloidal solution; b) ZIF-8 *in situ* grown in GO membranes.


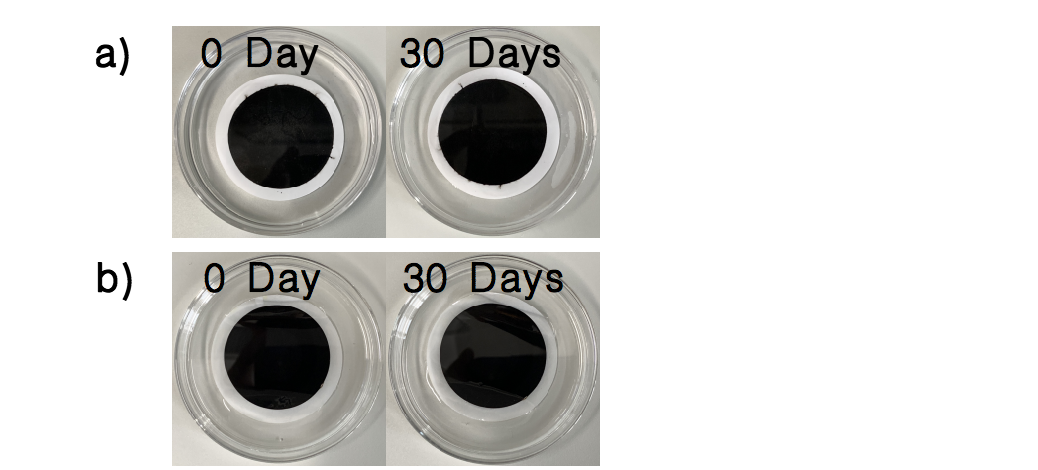


**Figure S12.** Photographs of ZIF-8-GO membrane after soaking in a) water and b) n-hexane for 30 days.


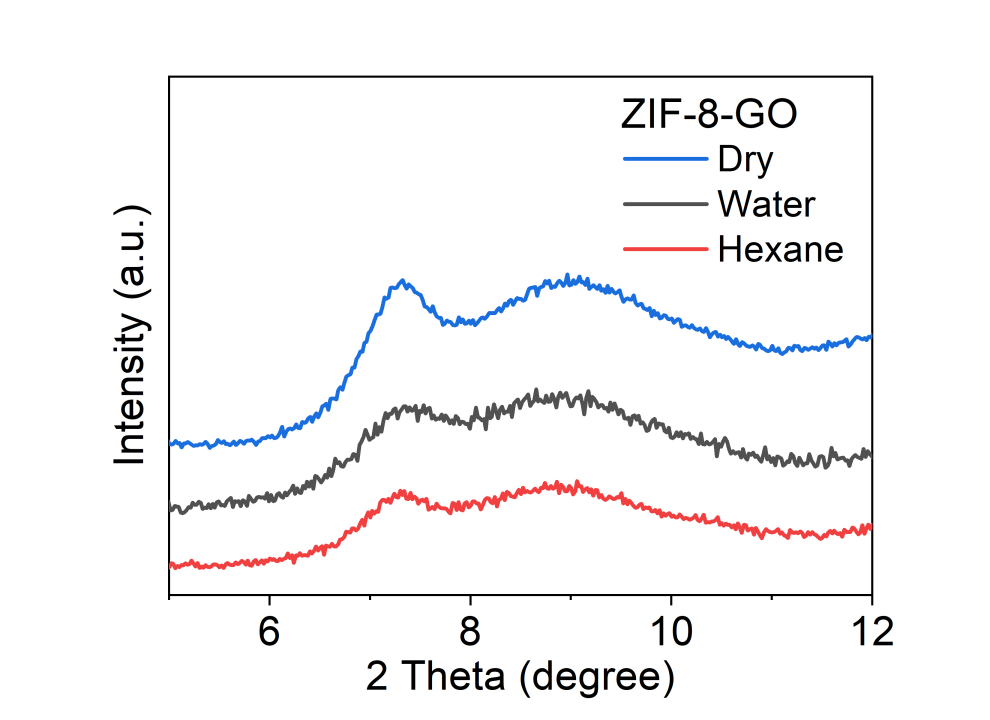


**Figure S13.** PXRD patterns of ZIF-8-GO in wet, dry and n-hexane immersion.


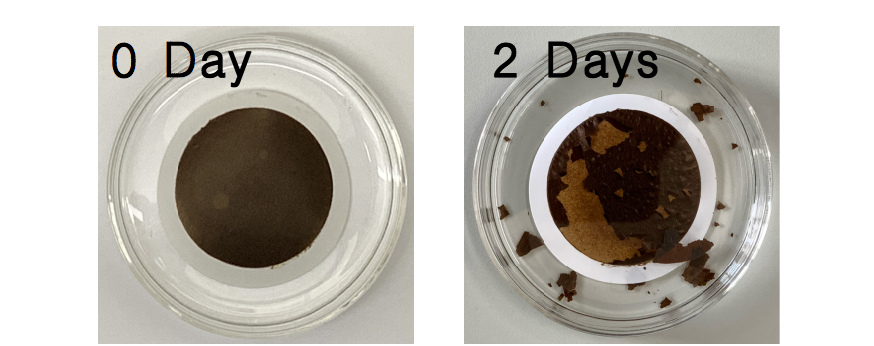


**Figure S14.** Photograph of GO membrane after soaking in water for two days.


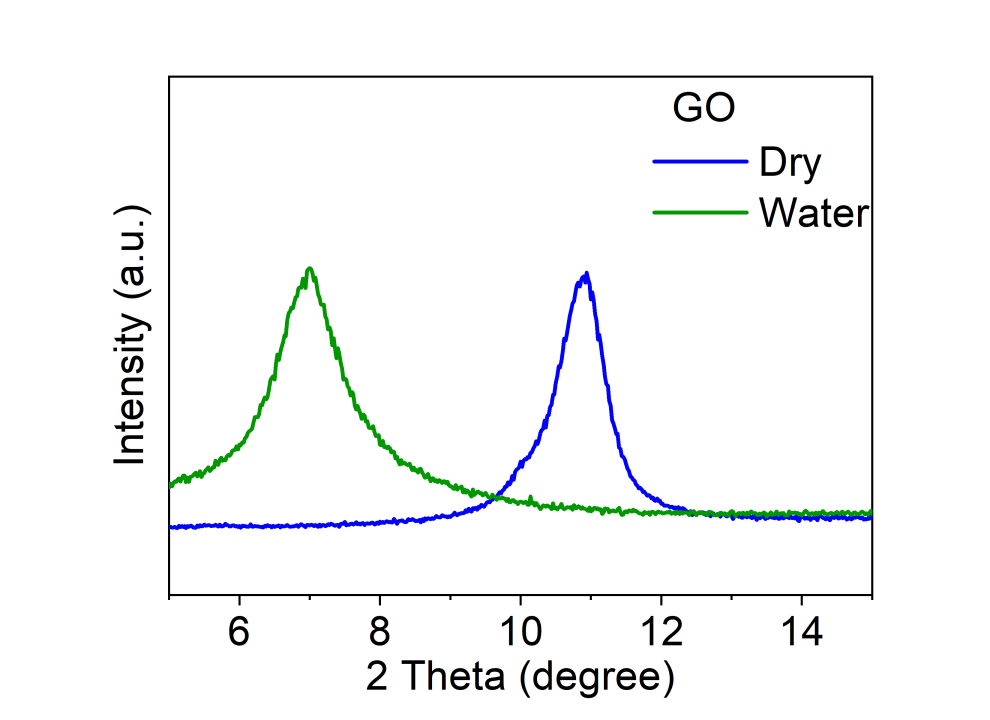


**Figure S15.** PXRD patterns of GO in the wet and dry states.


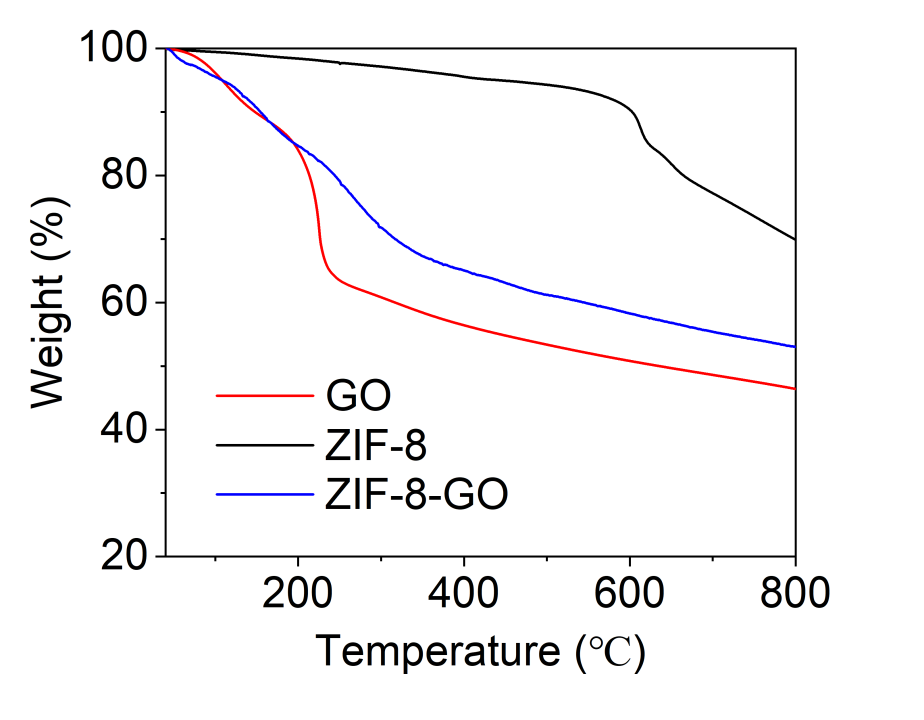


**Figure S16.** TGA curves of GO, ZIF-8 and ZIF-8-GO membrane under an ultra-high pure nitrogen atmosphere.

**
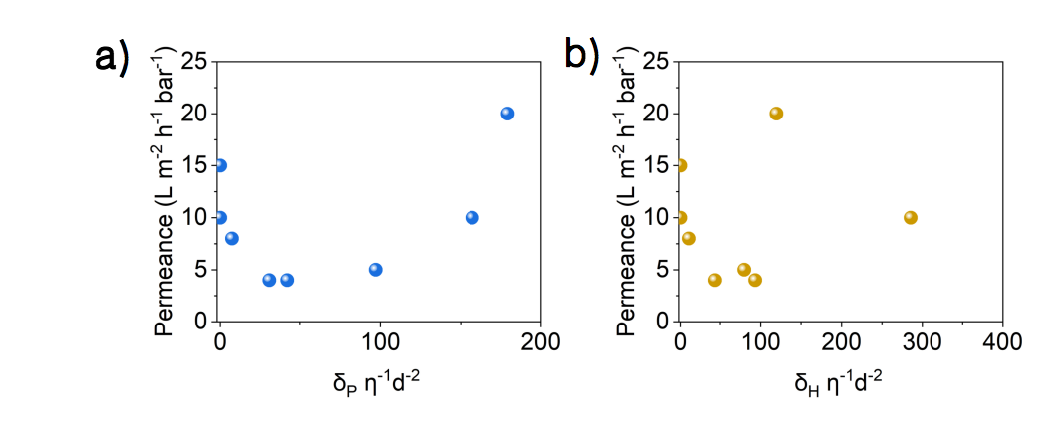
**

**Figure S17.** Permeability of organic solvents functioned (a) polarity and (b) hydrogen bond contributing to Hansen solubility parameter, viscosity and molecular diameter.

**
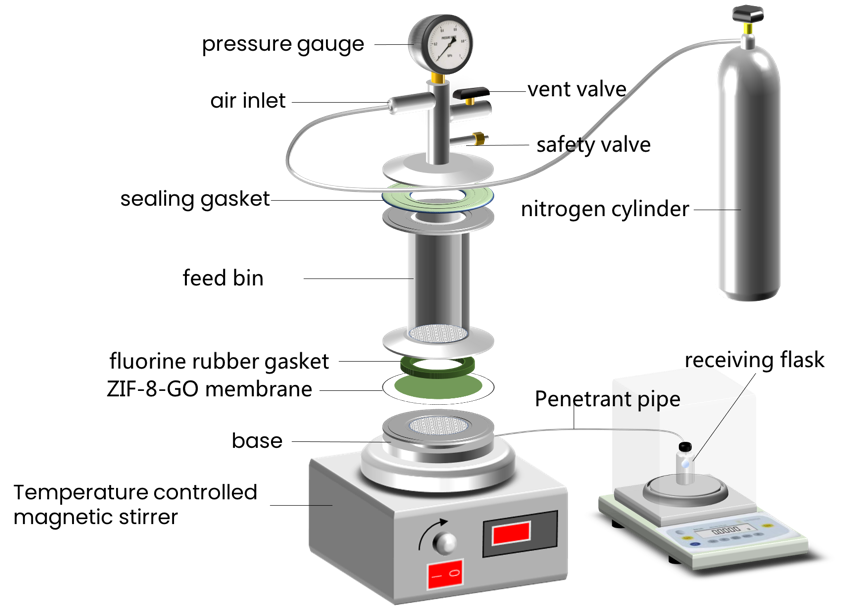
**

**Figure S18.** Schematic illustration of the dead-end filtration setup.

**
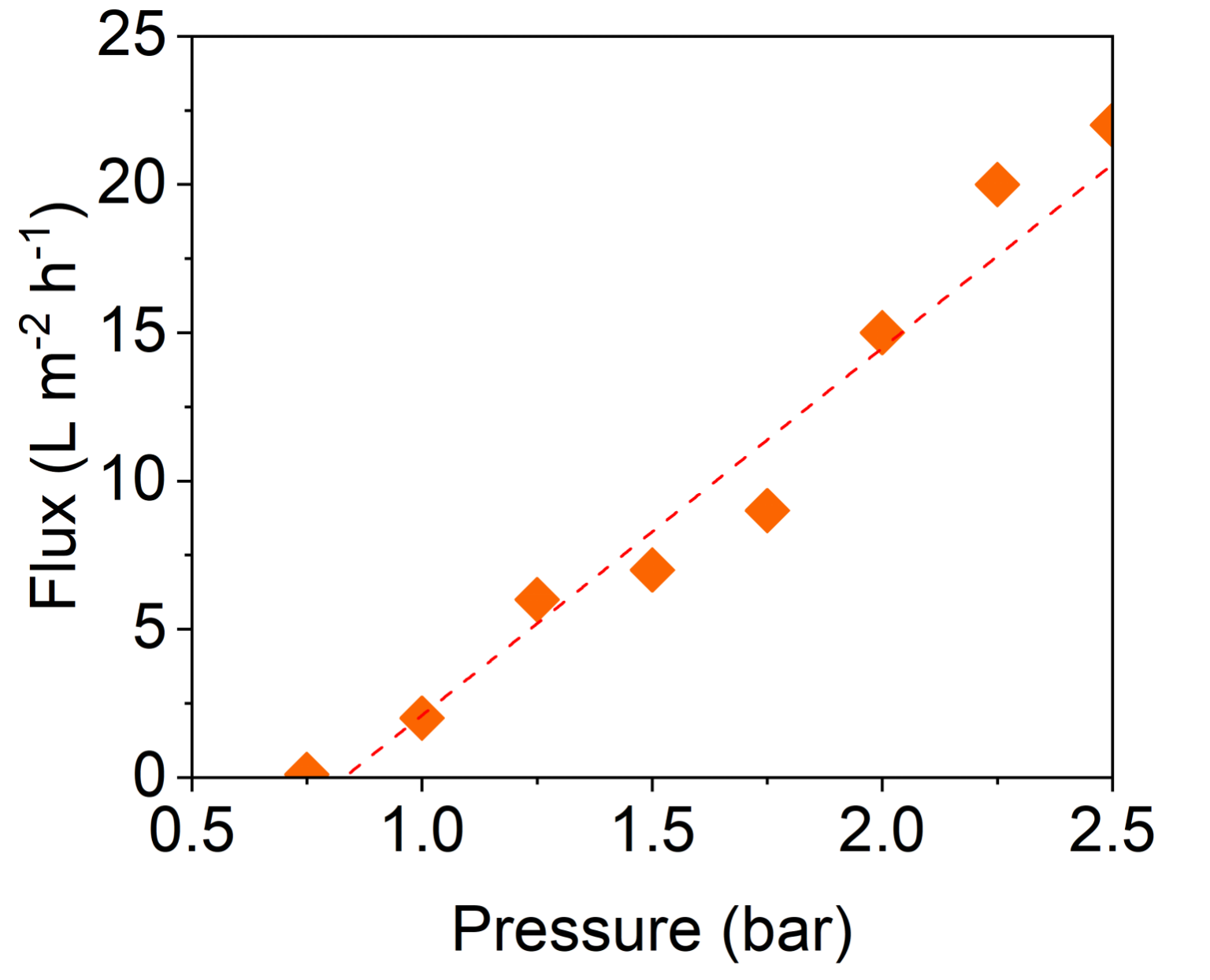
**

**Figure S19.** Flux of n-hexane under different pressures.


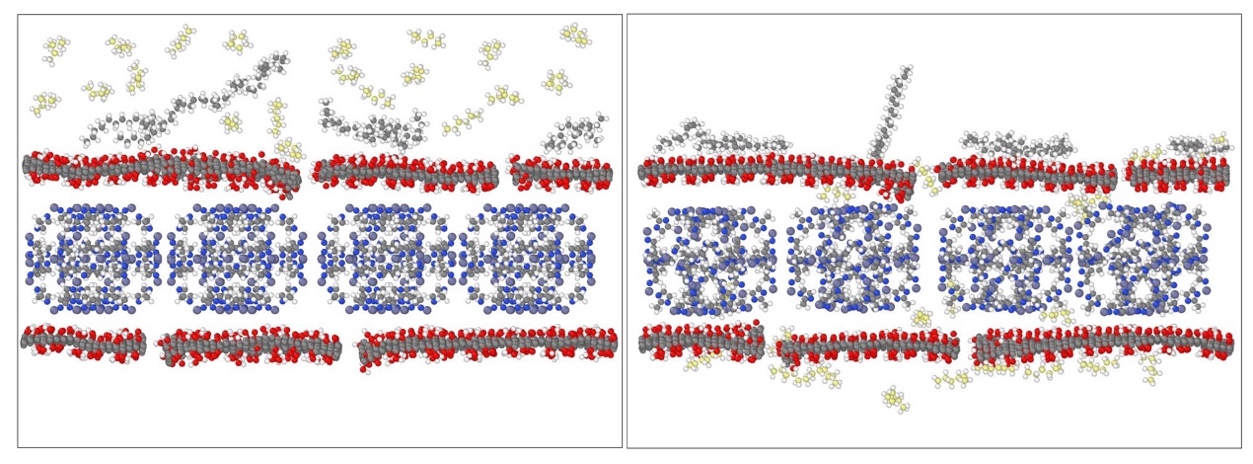


**Figure S20.** Schematic diagram of n-docosane and n-hexane passing through the ZIF-8-GO membrane.


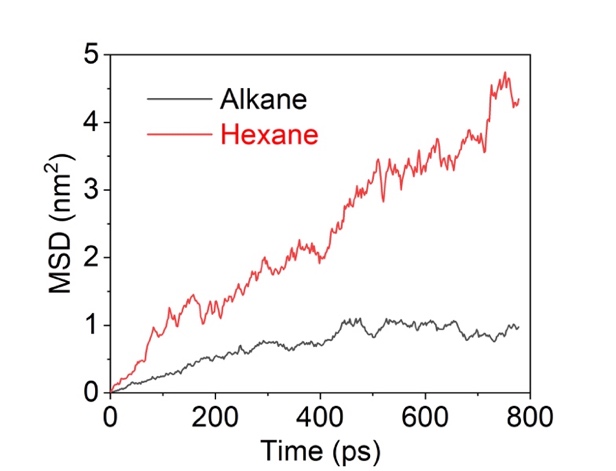


**Figure S21.** Dynamic diffusion simulation diagram of n-docosane and n-hexane in membrane separation process.

**
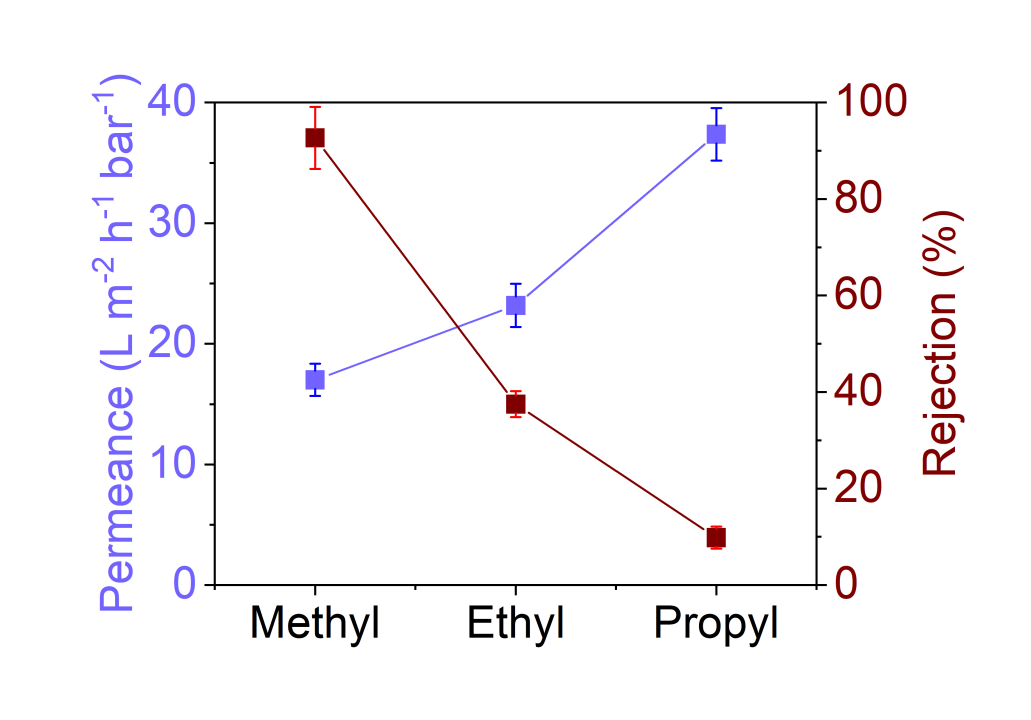
**

**Figure S22.** Permeance and rejection of quaternary ammonium salts with different groups (methyl, ethyl and propyl) inserted ZIF-8-GO membranes.


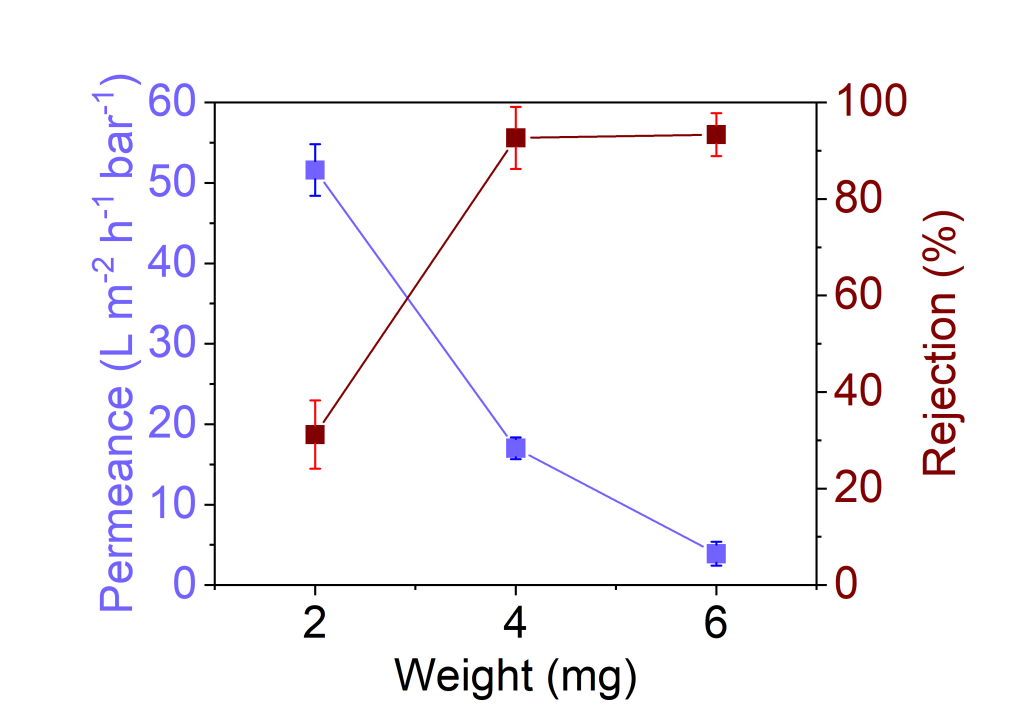


**Figure S23.** Permeance and rejection of ZIF-8-GO membranes with 2, 4 and 6 mg GO contents, separately.

**
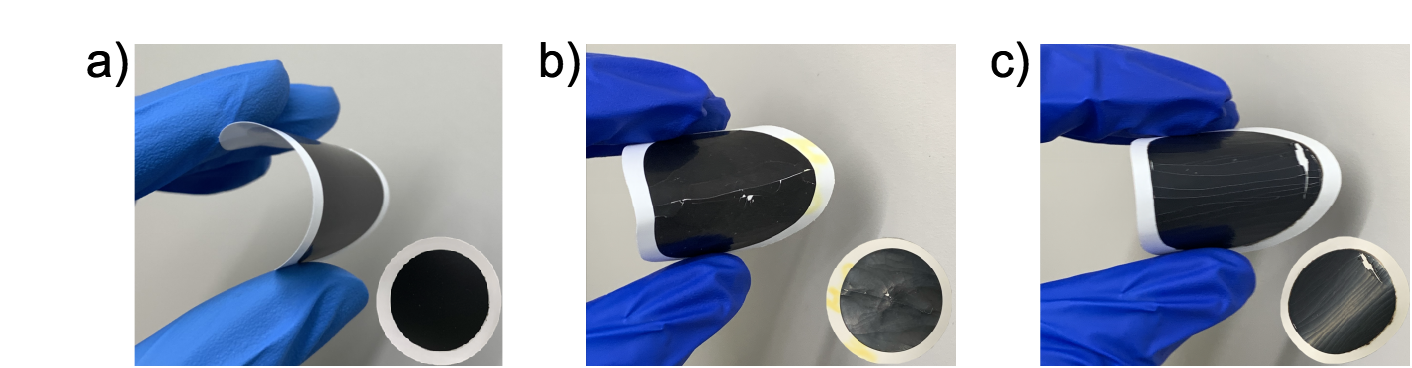
**

**Figure S24.** Photographs of quaternary ammonium salts with different groups, a) methyl, b) ethyl and c) propyl) inserted ZIF-8-GO membranes.


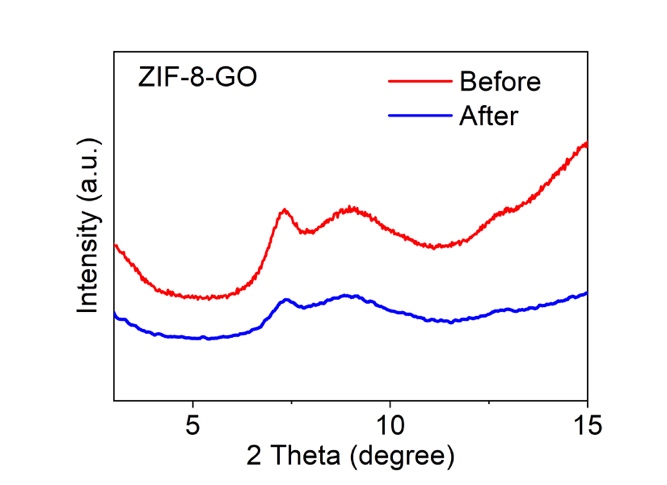


**Figure S25.** Comparison of XRD patterns of ZIF-8-GO membrane before and after 60 h test.


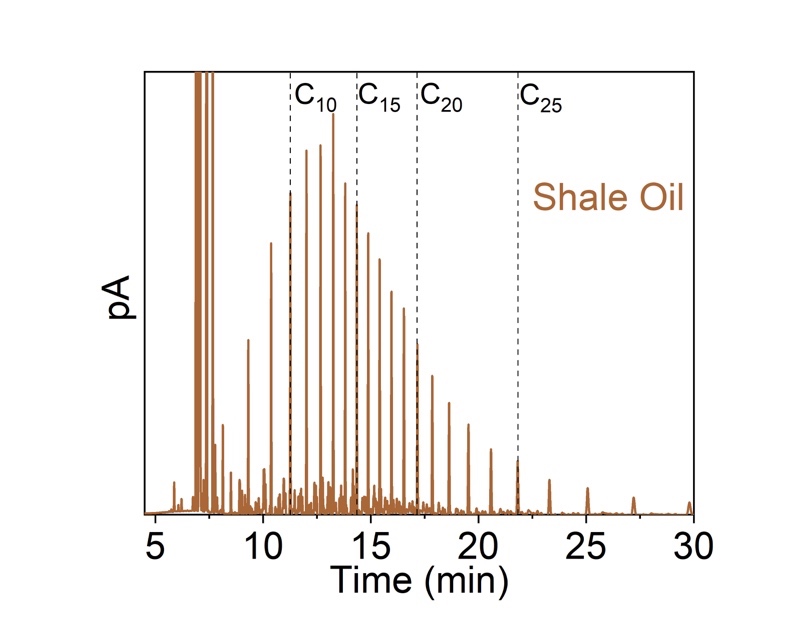


**Figure S26.** Gas chromatogram of shale oil calibrated by standard saturated hydrocarbon.

**Supplementary tables**

**Table S1.** The BET specific surface areas of GO, ZIF-8 and ZIF-8-GO membrane

|  | BET area (m^2^ g^−1^) |
| --- | --- |
| GO | 4 |
| ZIF-8 | 1675 |
| ZIF-8-GO | 97 |

**Table S2.** The characteristics of organic solvents involved in this experiment

|  | d (nm) | η (mPa·s) | δ (MPa^1/2^) | δ_P_ | δ_H_ |
| --- | --- | --- | --- | --- | --- |
| Acetone | 0.44 | 0.30 | 19.95 | 10.43 | 6.95 |
| Toluene | 0.59 | 0.55 | 18.32 | 1.40 | 2.00 |
| Hexane | 0.43 | 0.33 | 14.90 | 0 | 0 |
| Heptane | 0.45 | 0.39 | 15.20 | 0 | 0 |
| Methanol | 0.38 | 0.54 | 29.61 | 12.27 | 22.30 |
| DMF | 0.42 | 0.80 | 23.95 | 13.70 | 11.25 |
| Ethanol | 0.44 | 1.08 | 26.13 | 8.80 | 19.43 |
| THF | 0.63 | 0.46 | 19.46 | 5.70 | 8.00 |

**Table S3.** Detailed analysis of shale oil feed and permeate by the UPC_SIMDIS full range of simulated distillation systems

Feed

|  | Retention time (min) | Carbon number | Content (%) |
| --- | --- | --- | --- |
| 1 | 4.17-11.09 | 1-10 | 17.6 |
| 2 | 7.84-11.27 | 11-15 | 31.7 |
| 3 | 14.21-16.88 | 16-20 | 20.8 |
| 4 | 17.08-21.68 | 21-25 | 19.0 |
| 5 | 21.78-31.44 | 26-30 | 8.3 |
| 6 | ＞33.07 | ＞31 | 2.6 |

Permeate

|  | Retention time (min) | Carbon number | Content (%) |
| --- | --- | --- | --- |
| 1 | 4.17-11.09 | 1-10 | 44.9 |
| 2 | 7.84-11.27 | 11-15 | 41.4 |
| 3 | 14.21-16.88 | 16-20 | 11.7 |
| 4 | 17.08-21.68 | 21-25 | 1.1 |
| 5 | 21.78-31.44 | 26-30 | 0.8 |
| 6 | ＞33.07 | ＞31 | 0.1 |

**Table S4.** Summary of membrane performances

| Membrane | Solute | MWCO (g·mol^−1^) | Rejection (%) | Solvent | Permeance (L m^−2^ h^−1^ bar^−1^) | Pressure (bar) | Temperature (^o^C) | Ref. |
| --- | --- | --- | --- | --- | --- | --- | --- | --- |
| DUCKY-9 | Squalane | 415 | 94.4 ± 1.1 | Methanol  Toluene | 0.21 ± 0.01，0.47 ± 0.18 | 30 | 23 | ^[6]^ |
| D300-3h (PTA-OH) | Methyl orange | 327 | 95 (DMF) | Toluene | 10 | 2-5 | 30 | ^[7]^ |
| PIM-1 | HPB | 535 | 90 | Heptane | 18 | 13 | 30 | ^[8]^ |
| PDMS |  | 650 |  | Heptane | 1.1 |  |  | ^[9]^ |
| priamine-TA | styrene dimer | 235 | 90 | Heptane | 2.5 | 10 |  | ^[10]^ |
| Starmem122^®^(PI) | Sudan 408 | 464 | 78.7 | Toluene | 1.37 | 30 | 21 | ^[11]^ |
|  | PS oligomers | 270 | 90 | Toluene | 0.56 | 30 | 21 | ^[12]^ |
| PuraMem280(PI) |  | 280 | 90 | Toluene | 0.67 |  |  | ^[13]^ |
| PuraMemS600(PI) |  | 224 | 60 | Toluene | 0.7 |  |  |  |
| porphyrin/MPD | Sudan Blue II | 350 | 72.5 | Hexane | 28.7 | 2 | RT | ^[14]^ |
|  |  |  | 58 | Hexane | 8 | 2 | RT |  |
|  |  |  | 39 | Hexane | 1 | 2 | RT |  |
| LBL-aided β-CD | beta carotene | 537 | ≥90% | Hexane | 7 | 4 |  | ^[15]^ |
| α-Al_2_O_3_ |  | 410 |  | Hexane | 4.3 | 5 |  | ^[16]^ |
| PuraMem 280 | n-docosane | 310 | 72 ± 13 | Toluene | 0.51 ± 0.09 | 10 |  | ^[17]^ |
| SBAD-1 | n-docosane | 310 | 96 ± 1.2 | Toluene | 0.15 ± 0.03 | 10 |  |  |
| ONf-2 |  | 230 |  | Hexane | 2 | 10 |  |  |
| Puramem^®^ |  | 280 |  | Hexane | 0.23 | 35 | 25 | ^[18]^ |
| ZIF-8-GO | n-docosane | 310 | 94 ± 6.1 | Hexane | 16.4 ± 0.8 | 2 | 25 | **This work** |

**References**

[1] W. S. Hummers, Jr., R. E. Offeman, *J. Am. Chem. Soc.* **1958**, *80*, 1339.

[2] J. Cravillon, S. Münzer, S.-J. Lohmeier, A. Feldhoff, K. Huber, M. Wiebcke, *Chem. Mater.* **2009**, *21*, 1410.

[3] S. Plimpton, *J. Comput. Phys.* **1995**, *117*, 1.

[4] T. Weng, J. R. Schmidt, *J. Phys. Chem. A* **2019**, *123*, 3000.

[5] A. K. Rappe, W. A. Goddard, *J. Phys. Chem.* **2002**, *95*, 3358.

[6] N. C. Bruno, R. Mathias, Y. J. Lee, G. Zhu, Y.-H. Ahn, N. D. Rangnekar, J. R. Johnson, S. Hoy, I. Bechis, A. Tarzia, K. E. Jelfs, B. A. McCool, R. Lively, M. G. Finn, *Nat. Mater.* **2023**, *22*, 1540.

[7] S. Chisca, V.-E. Musteata, W. Zhang, S. Vasylevskyi, G. Falca, E. Abou-Hamad, A.-H. Emwas, M. Altunkaya, S. P. Nunes, *Science* **2022**, *376*, 1105.

[8] P. Gorgojo, S. Karan, H. C. Wong, M. F. Jimenez-Solomon, J. T. Cabral, A. G. Livingston, *Adv. Funct. Mater.* **2014**, *24*, 4729.

[9] M. Cook, L. Peeva, A. Livingston, *Ind. Eng. Chem. Res.* **2018**, *57*, 730.

[10] S.-H. Park, A. Alammar, Z. Fulop, B. A. Pulido, S. P. Nunes, G. Szekely, *Green Chem.* **2021**, *23*, 1175.

[11] S. Darvishmanesh, J. Degrève, B. Van der Bruggen, *Phys. Chem. Chem. Phys.* **2010**, *12*, 13333.

[12] S. Darvishmanesh, L. Firoozpour, J. Vanneste, P. Luis, J. Degrève, B. V. d. Bruggen, *Green Chem.* **2011**, *13*, 3476.

[13] H. Werhan, A. Farshori, P. Rudolf von Rohr, *J. Membr. Sci.* **2012**, *423-424*, 404.

[14] P. H. H. Duong, D. H. Anjum, K.-V. Peinemann, S. P. Nunes, *J. Membr. Sci.* **2018**, *563*, 684.

[15] X. Li, C. Li, K. Goh, T. H. Chong, R. Wang, *J. Membr. Sci.* **2022**, *652*, 120466.

[16] H. J. Lee, S.-J. Kim, Y. Kim, H. Park, Y.-I. Park, S. E. Nam, *Ceram. Int.* **2021**, *47*, 34020.

[17] K. A. Thompson, R. Mathias, D. Kim, J. Kim, N. Rangnekar, J. R. Johnson, S. J. Hoy, I. Bechis, A. Tarzia, K. E. Jelfs, B. A. McCool, A. G. Livingston, R. P. Lively, M. G. Finn, *Science* **2020**, *369*, 310.

[18] A. R. S. Teixeira, J. L. C. Santos, J. G. Crespo, *J. Membr. Sci.* **2014**, *470*, 138.
